# Natural Products from Herbal Medicine Self‐Assemble into Advanced Bioactive Materials

**DOI:** 10.1002/advs.202403388

**Published:** 2024-07-21

**Authors:** Xiaohang Guo, Weikang Luo, Lingyu Wu, Lianglin Zhang, Yuxuan Chen, Teng Li, Haigang Li, Wei Zhang, Yawei Liu, Jun Zheng, Yang Wang

**Affiliations:** ^1^ School of Medicine Hunan University of Chinese Medicine Changsha 410208 China; ^2^ Institute of Integrative Medicine Department of Integrated Traditional Chinese and Western Medicine Xiangya Hospital Central South University Changsha 410008 China; ^3^ Center for Interdisciplinary Research in Traditional Chinese Medicine Xiangya Hospital Central South University Changsha 410008 China; ^4^ National Clinical Research Center for Geriatric Disorders Xiangya Hospital Central South University Changsha 410008 China; ^5^ College of Traditional Chinese Medicine Hunan University of Chinese Medicine Changsha 410208 China; ^6^ Beijing Normal University‐Hong Kong Baptist University United International College Zhuhai 519087 China; ^7^ Hunan key laboratory of the research and development of novel pharmaceutical preparations Changsha Medical University Changsha 410219 China; ^8^ College of Integrated Chinese and Western Medicine Hunan University of Chinese Medicine Changsha 410208 China

**Keywords:** bioactive materials, natural products, self‐assembly, strategy

## Abstract

Novel biomaterials are becoming more crucial in treating human diseases. However, many materials require complex artificial modifications and synthesis, leading to potential difficulties in preparation, side effects, and clinical translation. Recently, significant progress has been achieved in terms of direct self‐assembly of natural products from herbal medicine (NPHM), an important source for novel medications, resulting in a wide range of bioactive supramolecular materials including gels, and nanoparticles. The NPHM‐based supramolecular bioactive materials are produced from renewable resources, are simple to prepare, and have demonstrated multi‐functionality including slow‐release, smart‐responsive release, and especially possess powerful biological effects to treat various diseases. In this review, NPHM‐based supramolecular bioactive materials have been revealed as an emerging, revolutionary, and promising strategy. The development, advantages, and limitations of NPHM, as well as the advantageous position of NPHM‐based materials, are first reviewed. Subsequently, a systematic and comprehensive analysis of the self‐assembly strategies specific to seven major classes of NPHM is highlighted. Insights into the influence of NPHM structural features on the formation of supramolecular materials are also provided. Finally, the drivers and preparations are summarized, emphasizing the biomedical applications, future scientific challenges, and opportunities, with the hope of igniting inspiration for future research and applications.

## Graphical abstract

Illustrative overview of the application and characterization of self‐assembly of natural products of herbal medicine (NPHM) into advanced bioactive materials. Created with BioRender.com.

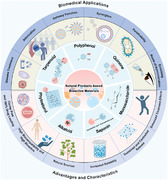

## 1 Introduction

Responding to the changing spectrum of human diseases and the unsatisfactory results of existing drug treatments, the pursuit of human health and quality of life are the most pressing responsibilites of the modern world and an unwavering focal point fot a better future. Over the past thousands of years, herbal medicines have been playing an important role in human health. With scientific advancements, a range of highly valuable medicinal natural products from herbal medicine (NPHM) have been extracted, which are rich and unique in terms of structural skeleton and stereochemistry, allowing for the effective combination of drug targets, regulation of physiological processes, and treatment of a variety of diseases. Therefore, NPHM have become one of the most valuable source of new drugs and significantly contributed to human therapeutics,^[^ ^1^ ^]^ such as artemisinin for malaria,^[^ ^2^ ^]^ paclitaxel for tumors,^[^ ^3^ ^]^ morphine for anesthesia,^[^ ^4^ ^]^ aspirin for inflammation,^[^ ^5^ ^]^ and many more. Unfortunately, a significant proportion of approximately 90% of NPHM are compelled to be excluded from new drug screening processes because of their inadequate water solubility, limited bioavailability, and low stability when applied in practical clinical settings.^[^ ^6^ ^]^ Therefore, fully utilization and exhibition of the biological activity of NPHM, an important drug resource endowed by nature to create greater value for human society, is an important challenge for modern society.

Cross‐fertilization between medicine, supramolecular chemistry, and materials has led to new ideas for innovative technologies. Interestingly, researchers discovered that the privileged structure of NPHM, not only exhibits bioactivity but also enables self‐assembly, which directly contributes to the advancement in its value.^[^ ^7^ ^]^ Self‐assembly refers to the spontaneous formation of ordered structures by chaotic fundamental elements driven by non‐covalent interactions.^[^ ^8^ ^]^ NPHM can self‐assemble into a range of supramolecular bioactive materials without the addition of a carrier or modification by covalent bonds, such as nanoparticles,^[^ ^9^ ^]^ micelles,^[^ ^10^ ^]^ gels^[^ ^11^ ^]^ When the NPHM‐based supramolecular bioactive materials reaches a particular environment, non‐covalent interactions dissociate responsively, causeing NPHM to exert their medicinal effects. Overcoming the limitations of NPHM monomers, the NPHM‐based supramolecular bioactive materials have achieved transcendence in functionality and bioactivity in terms of: I) enhanced solubility and stability,^[^ ^12^ ^]^ II) penetration of biological barriers,^[^ ^13^ ^]^ III) slow‐release^[^ ^14^ ^]^ and smart responsive release,^[^ ^15^ ^]^ IV) synergism,^[^ ^16^ ^]^ and toxicity reduction,^[^ ^17^ ^]^ etc. Furthermore, NPHM has also brought new perspectives to the selection of building members in the biomaterials field. Compared with others, the NPHM‐based supramolecular bioactive materials have the following obvious advantages: I) they are derived from renewable resources to achieve sustainable development, high biocompatibility and high biodegradability; II) simple to prepare, in line with easy production, green chemistry, and environmental friendliness; and III) composed of NPHM with potent pharmacological activities,^[^ ^18^ ^]^ capable of modulating pathological processes and treating various types of diseases; and IV) exhibit drug self‐delivery and realizing a 100% high drug loading. Therefore, the NPHM‐based supramolecular bioactive materials not only overcome the limitations of NPHM monomers, but also present an important new class of bioactive materials to mankind as compared to non‐bioactive nanomaterials used in traditional biomedical applications, and are emerging as new concepts, technologies, and frameworks in the field of biomaterials. It is believed that the NPHM‐based supramolecular bioactive materials are rising stars with a bright and irreplaceable future, showing great potential for clinical application and translation.

As a novel strategy, a systematic and complete theoretical system is essential and plays a guiding role. However, despite significant investments in resources, researchers still rely on luck to identify the self‐assembly behavior of NPHM after conducting various tests. Existing literature clearly describes the present state of research in this field, but the laws governing its occurrence and evolution have not been summarized. Thus, an immediate requirement exists for a structured and methodological guide for this active study. This review proposes NPHM‐based self‐assembly materials as novel, efficient, and advanced bioactive nanomaterials and for the first time provides a systematic and comprehensive overview of specific self‐assembly strategy guidelines for different types of NPHM, with the expectation of saving costs and facilitating future research. In this paper, we first introduce the biological activity and limitations of NPHM. Then, we discuss the advantages of NPHM‐based supramolecular bioactive materials. In the following sections, NPHM will be classified into the following seven categories according to the assembly mechanism: polyphenols, quinones, monosaccharides, saponins, alkaloids, phytosterols, and terpenoids. We will focus on the unique self‐assembly strategies of each category of NPHM, including the influence of the structural features of NPHM on the self‐assembly mechanism, and the characteristics and functions of the supramolecular products. Subsequently, the supramolecular drivers and methods of preparations of NPHM‐based supramolecular bioactive materials are summarized. The last part highlights the medical applications of NPHM self‐assembly technology and comments on the scientific challenges and future directions of the NPHM‐based supramolecular biomaterials in academic research and their practical applications.

## 2 Origins and Advantages of NPHM‐Based Supramolecular Bioactive Materials

### 2.1 Origins of Natural Products from Herbal Medicine

Herbal medicines have been utilized in medicine for millennia throughout human history. An illustrative example is the 16th‐century Compendium of Materia Medica, which provides information on ≈1900 distinct medicinal plants and their respective therapeutic attributes. Various components of natural herbs such as leaves, flowers, buds, pollen, fruits, skins, seeds, stems, roots, rhizomes, bark, and even entire herbs can be utilized for medical applications, each possessing distinct therapeutic qualities.^[^ ^19^ ^]^

With the advancement of science and technology, researchers have extracted nmuerous natural products (secondary metabolites)^[^ ^20^ ^]^ from herbal medicine (NPHM) and herbal medicines are considered as one of the important sources of natural products.^[^ ^21^ ^]^ NPHM are the secondary metabolites of herbs. Morphine, an alkaloid extracted from the herb opium, is considered to be the first NPHM discovered in human history, and was subsequently widely used as an anesthetic and analgesic drug,^[^ ^22^ ^]^ starting the human exploration of natural products in plants. Subsequent studies have revealed the therapeutic potential of various natural products, thus establishing them as one of the important sources of medicines for mankind.^[^ ^23^ ^]^ Notable examples include artemisinin from Artemisia annua,^[^ ^24^ ^]^ camptothecine from the camptotheca acuminata,^[^ ^25^ ^]^ paclitaxel from the bark of Picea abies,^[^ ^26^ ^]^ aspirin from Willow Bark, and metformin from Galega officinalis,^[^ ^27^ ^]^ among others. Therefore, NPHM has been and will continue to be a great contributor to human health and well‐being.

### 2.2 Advantages and Limitations of Natural Products from Herbal Medicine

Specifically, NPHM have the following five advantages: I) Natural and renewable: As NPHM are sourced from nature, they represent a renewable resource.^[^ ^28^ ^]^ With the increasing global demand for energy and rising awareness of environmental protection, utilizing renewable resources is a sustainable and environmentally friendly option.^[^ ^29^ ^]^ II) The vast array and diverse nature of sources is remarkable: Globally, it is approximated that ≈70000 herbal medicines are utilized for medicinal applications.^[^ ^30^ ^]^ Furthermore, according to the COCONUT (Collection of Open Natural Products) database, an impressive tally of over 400000 natural products has been identified, highlighting the remarkable diversity and richness of its contents.^[^ ^31^ ^]^ Consequently, NPHM offers an exceptionally broad range of options for selection. III) High biocompatibility and biodegradability.^[^ ^32^ ^]^ IV) Extensive scaffold diversity and intricate structural complexity: NPHM is the privileged structures formed by nature over a long‐term evolution and contains unique chemical spaces,^[^ ^33^ ^]^ such as the enone structure, a distinctive structural motif for biologically potent natural products and drugs,^[^ ^34^ ^]^ alongside the 3,3′‐pyrrolidinyl spiroindole scaffold,^[^ ^35^ ^]^ numerous oxygen‐containing substituents, ample stereochemical centers,^[^ ^23^ ^]^ and the sp3 carbons.^[^ ^36^ ^]^ This chemical diversity and complexity lay the foundation for their specific and abundant pharmacological activities.^[^ ^36^ ^]^ (V) Powerful pharmacological activity: NPHM is rich in pharmacological activities that bind biological targets with high selectivity and specificity on a mechanism‐of‐action basis.^[^ ^37^ ^]^Therefore, NPHMs are the most important source of new drugs, and currently, multiple NPHMs have been approved by the National Medical Products Administration and the U.S.  Food and Drug Administration as anticancer drugs, cardiovascular and cerebrovascular disease drugs, anti‐infective drugs, central nervous system disease drugs, digestive disease drugs, immunomodulatory drugs, and metabolism drugs.^[^ ^38^ ^]^

Despite the significant advantages and uniqueness brought by the complex chemical structure of NPHM, approximately 70% of them exhibit hydrophobic characteristics leading to their low solubility, which in turn affects their bioavailability and limits their therapeutic potential. Currently, up to 90% of pharmacologically active NPHMs are excluded from the new drug screening processes.^[^ ^6^ ^]^ Therefore, the development of new approaches to improve the bioavailability of NPHM is essential to realize its full potential in the treatment of human diseases.

In conclusion, realizing the advantages of using NPHM while avoiding its disadvantages has become the eager pursuit for researchers to promote human health. Thus, NPHM‐based self‐assembled supramolecular materials provide new perspectives for researchers.

### 2.3 Particularities of NPHM‐Based Supramolecular Bioactive Materials

Emerging as a cutting‐edge class of materials in materials science, NPHM‐based supramolecular bioactive materials are gaining significant attention. Researchers have discovered that NPHM monomers possess the remarkable ability to self‐assemble into nanoparticles, gels, and diverse supramolecular structures, all achieved through non‐covalent interactions among the monomers—without requiring any structural alterations or covalent bonding. What unique properties do these NPHM‐based supramolecular bioactive materials possess that highlight new research directions in materials science? This section will provide a general overview, exploring the key properties, and value of these materials.

**Figure 1:**
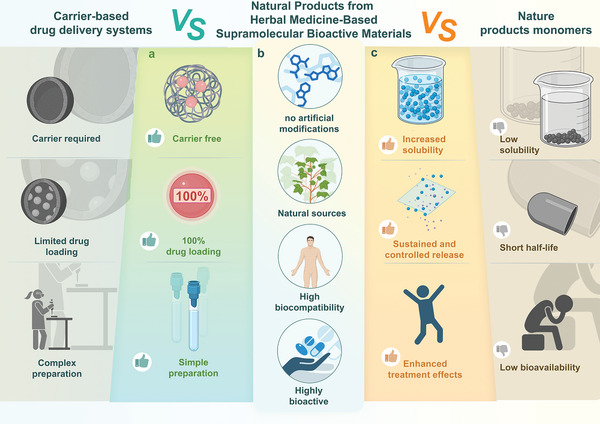
Schematic representation of the particularities of NPHM‐based supramolecular bioactive materials. a) The advantages of NPHM‐based supramolecular bioactive materials over carrier‐based systems, including carriers‐free, a 100% drug loading rate, and simplicity of preparation. b) As building blocks, NPHMs impart unique properties to NPHM‐based supramolecular bioactive materials, such as their avoidance of artificial modifications, natural origins, high biocompatibility, and diverse pharmacological activities. c) The advantages of NPHM‐based supramolecular bioactive materials over NPHM monomers include increased solubility, sustained and controlled release, and enhanced treatment effects. Created with BioRender.com.

To clarify this issue, we need to discuss it from two perspectives and compare two objects. The first comparison is between NPHM‐based supramolecular materials and carrier‐based delivery systems (**Figure** **1**a). In the field of drug delivery, technologies utilizing delivery carriers to administer various drugs with suboptimal bioavailability are becoming more mature, offering increased stability and enhanced functionality. However, some issues still need to be optimized, and NPHM‐based bioactive materials have shown superior performance in addressing these challenges.

First, patients face difficulties in metabolism and eliminating these carrier substances, such as inorganic porous carriers, polymers, etc., which can be challenging and pose certain risks.^[^ ^39^ ^]^ In contrast, the direct self‐assembly of pharmacologically active NPHMs into NPHM‐based bioactive materials allows for direct drug self‐delivery, thereby eliminating the dependence on carriers.

Second, the drug loading capacity is inherently constrained by the spatial structure and properties of the carrier material, posing a significant limitation. This limitation poses a challenge, as to achieve sufficient drug concentration in the target area with delivery carriers having limited capacity, increased usage is required, potentially leading to undesirable side effects from the carriers themselves. Conversely, NPHM‐based bioactive materials boast a remarkable drug‐loading rate of up to 100%.

Third, the cost of preparation is a crucial factor in clinical translation. The isolation, extraction, and preparation of certain delivery vehicles, like extracellular vesicles, encounter challenges, including intricate processes and barriers to large‐scale, cost‐effective production. In stark contrast, the preparation of NPHM‐based bioactive materials is straightforward. It holds the potential to facilitate low‐cost, large‐scale production, thereby advancing their practical clinical applications in the future.

The second comparison revolves around NPHM‐based supramolecular materials and their respective monomers (Figure 1c). From the previous summary, it is clear that while NPHM monomers possess powerful pharmacological activities, their therapeutic potential is limited by poor water solubility, short half‐life, low bioavailability, among others. Forming NPHM‐based supramolecular bioactive materials realizes the surpassing of NPHM monomers from the following aspects.

First, NPHM‐based supramolecular bioactive materials significantly improve solubility,^[^ ^40^ ^]^ thereby directly influencing the bioavailability of NPHM. This improvement is significantly attributed to the increased surface area of NPHM self‐assembled nanoproducts, which accelerates drug dissolution in liquids. Consequently, both the dissolution rate and saturation solubility experience a remarkable increase.^[^ ^41^ ^]^ For example, the solubility of the NPHM baicalin increased 4500‐fold when encapsulated by micelles formed through the self‐assembly of another NPHM, glycyrrhizic acid (GA).^[^ ^12^ ^]^

Second, NPHM‐based supramolecular bioactive materials possess drug‐controlled release properties, enabling the slow and sustained release of NPHMs over an extended period. This ensures prolonged efficacy while minimizing the necessity for frequent administrations.^[^ ^42^ ^]^ As an example, Zheng et al. formulated a hydrogel composed of rhein, an anti‐inflammatory NPHM, which gradually disseminates rhein in a 3D gel matrix. This controlled‐release mechanism significantly enhanced the anti‐neuroinflammatory efficacy compared to the rhein monomer alone.^[^ ^11^ ^]^ Furthermore, the unique properties of the NPHM‐based materials endow them with functionalities that NPHM monomers lack, including self‐healing capabilities, injectability, and so on. Owing to the above features, NPHM‐based supramolecular bioactive materials significantly enhance the bioactivities of NPHM monomers and exhibit more exceptional therapeutic potential. Furthermore, NPHM‐based supramolecular bioactive materials, which directly self‐assemble from NPHM, inherit the advantages of NPHM, encompassing natural sources, no artificial modifications, high biocompatibility, and diverse pharmacological activities (Figure 1b). In summary, these materials not only transcend the limitations of NPHM, maximizing its utilization but also introduce novel building blocks with significant pharmacological potential to the materials science community, positioning them as emerging stars. The synergistic combination of self‐assembly technology and NPHM has fostered mutual enhancement and advancement, heralding further progress in the future.

Having outlined the this material's fundamental characteristics, we will delve deeper into the specifics and elaborate on the seven principal categories of NPHMs that are most representative and extensively studied in the realm of NPHM self‐assembly, emphasizing their assembly methodologies and research advancements.

## 3 Strategy Guides for 7 Classes of NPHM Self‐Assembling to Bioactive Material

Exploring the self‐assembly behavior of NPHM is extremely challenging and often relies on the experience and serendipity of researchers. This underscores the n systematic studies on the regularity of NPHM self‐assembly and the need for structured strategies. Therefore, this section aims to provide the first systematic guidance strategy for NPHM self‐assembly material from a regularity perspective.

Initially, common factors such as temperature and pH were considered, but it was found that the response to these conditions varies due to the complex structure of NPHM. The different functional groups result in a variety of self‐assembly modes and products under supramolecular driving forces. Therefore, this review attempts to find commonalities among these differences and ultimately summarizes the classification of NPHM into seven major groups – polyphenols, monosaccharides, saponins, quinones, alkaloids, phytosterols and terpenoids – based on the structural similarities that lead to similar self‐assembly behaviors. The next sections go beyond focusing on these commonalities and emphasize the unique self‐assembly strategies of each class. This classification approach aims to provide a clear framework and insights for future studies, deepen the understanding of the self‐assembly behavior of various NPHM, and offer a theoretical basis for developing targeted self‐assembly strategies.

### 3.1 Self‐assembly of Polyphenols

Natural polyphenols are widely found in many plants and fruits (e.g., green tea, citrus fruit, coffee,^[^ ^43^ ^]^ traditional Chinese medicine cutellaria baicalensis Georgi root, etc.) and have a variety of biological activities including antioxidant,^[^ ^44^ ^]^ anti‐aging,^[^ ^45^ ^]^ anti‐bacterial,^[^ ^46^ ^]^ anti‐cancer, anti‐inflammatory, and cardioprotective.^[^ ^47^ ^]^

Structurally, natural polyphenols are defined as substances containing one or more phenolic hydroxyl groups attached to one or more aromatic rings. In this section, we will classify polyphenols based on whether their key self‐assembling structures are catechols or pyrogallol. The commonalities and properties of self‐assembly for each class will be separately summarized.

#### 3.1.1 Self‐assembly of Polyphenols with Catechol or Pyrogallol Structure

**Figure 2:**
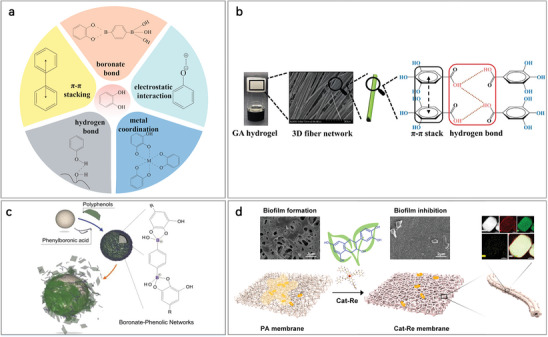
Schematic representation of the self‐assembly of polyphenols with catechol or pyrogallol structure: from five supramolecular drivers to three major self‐assembly strategies with NPHM, phenylboronic acid and metal ions respectively. a) Supramolecular interactions of polyphenols with catechol or pyrogallol structure. b) Mechanism of gallic acid self‐assembly via π – π stacking and hydrogen bonding to form hydrogels. Reproduced with permission.^[^ ^52^ ^]^ Copyright 2022, Wiley. c) Formation of boronate‐phenolic networks (BPNs) and preparation of BPNs films by polyphenols and boric acid via boronic acid bonding on the surface of particle templates. Reproduced with permission.^[^ ^53^ ^]^ Copyright 2015, Wiley. d) Polyphenol catechins co‐assembled with Re^3+^ to form MPNs with bioadhesive ability to cover the surface of polyamide membranes and provide antimicrobial effects. Reproduced with permission.^[^ ^54^ ^]^ Copyright 2020, ACS.

Polyphenols containing the structure of catechol or pyrogallol, e.g., quercetin, gallic acid, tannins, etc., have a wide range of biological activities such as anti‐neuroinflammatory,^[^ ^48^ ^]^ hypotensive, anti‐hyperlipidaemic,^[^ ^49^ ^]^ and anticancer.^[^ ^50^ ^]^ The catechol or pyrogallol structure is crucial both for the chemical properties of polyphenols and their self‐assembly strategies. These structures promote self‐assembly primarily through five major supramolecular interaction forces: hydrophobic interactions, electrostatic interactions, *π*–*π* stacking, hydrogen bonding, boronate bonding, and metal coordination (**Figure** **2**a).^[^ ^51^ ^]^ Next, we will first summarize three assembly strategies of polyphenols with catechol or pyrogallol, respectively, including I) direct self‐assembly; II) metal−phenolic networks (MPNs); and III) boronate−phenolic networks (BPNs). Finally, three major properties common to the above three strategies will be summarized.

##### Self‐Assembly of Polyphenols with NPHM

The polyhydroxy and benzene ring structures play a key role in the direct self‐assembly process of polyphenols through *π*–*π* stacking and hydrogen bonding driven predominantly to form products represented by hydrogels and nanoparticles.^[^ ^55^ ^]^ Gallic acid, a polyphenol extracted from plants such as grapes, tea, and oak,^[^ ^56^ ^]^ can self‐assemble via π – π stacking and hydrogen bonding into hydrogels with injectability, thermal reversibility, self‐healing, and antimicrobial activity (Figure 2b). However, gallic acid hydrogels are limited by easy degradation and low viscosity.^[^ ^52^ ^]^ In order to further solve the problem, the team of Hu et al. made an attempt at multicomponent co‐assembly. With further research, they found that gallic acid and resveratrol, another natural polyphenol small molecule, can co‐assemble to form a two‐component co‐assembled hydrogel through hydrogen bonding and *π*–*π* interactions. The experimental results showed that the two‐component hydrogel was superior in terms of both apparent properties (strength and viscosity) and biological functions (scavenging of lipopolysaccharide‐induced reactive oxygen species (ROS) and macrophage‐produced nitric oxide), leading to enhanced antimicrobial efficacy when used as a wound dressing.^[^ ^57^ ^]^ This reminds us that constructing multicomponent co‐assembly hydrogels is beneficial in improving the mechanical properties and therapeutic effects of gels, which may be a future research direction.

##### Self‐Assembly of Polyphenols with Boronic Acid: Boronate−Phenolic Networks (BPNs)

BPNs are formed because of the transition of boron from sp^2^ to tetrahedral sp^3^ hybridization in boronic acid, followed by a reaction with cis‐diols on polyphenols to form cyclic boronic esters. The stability of BPNs is influenced by pH, specifically, they are stabilized only when the pH exceeds the pKa of boric acid, rendering the materials constructed with BPNs pH‐responsive.^[^ ^58^ ^]^ Guo et al. synthesized thin films of BPNs utilizing phenylboronic acid and tannic acid on the surface of a spherical template. Following this, the template was removed to yield BPNs capsules (Figure 2c).^[^ ^53^ ^]^ Additionally, BPNs can be formed between polyphenols containing a catechol structure and other compounds with adjacent hydroxyl groups. For example, catechins and isoguanosine can be co‐assembled via dynamic phenylborate diester bonds to form injectable hydrogels for the treatment of Human papillomavirus‐associated oral squamous cell carcinoma.^[^ ^59^ ^]^

##### Self‐Assembly of Polyphenols with Metal Ions: Metal−Phenolic Networks (MPNs)

**Figure 3:**
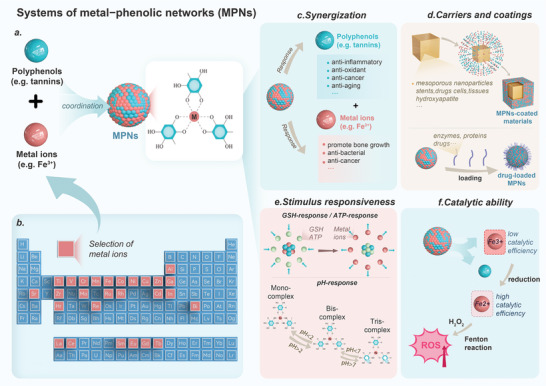
Schematic representation of the formation and properties of metal−phenolic networks (MPNs). a) Schematic representation of polyphenols and metal ions forming MPNs by coordination. b) Selected range of metal ions that have been reported to construct MPNs, indicated by the red squares of the elemental cycle. Reproduced with permission.^[^ ^58^ ^]^ Copyright 2022, ACS. c) Polyphenols and metal ions exerting a synergistic biological effect. d) MPNs used in the application of delivery carriers and coatings. e) pH, glutathione (GSH), and ATP responsiveness of MPNs. f) Schematic representation of the catalytic capacity of MPNs.

MPNs are formed through coordination‐driven self‐assembly involving metal ions and polyphenols (**Figure** **3**a).^[^ ^60^ ^]^ Phenolic compounds act as electron donors, while transition metals act as electron acceptors. This interaction occurs when hydroxyl oxygen atoms from catechol or pyrogallol donate electron pairs to empty orbitals in the metal ions, resulting in the formation of metal‐phenolic coordination when multiple phenolic ligands contribute nonbonding electron pairs.^[^ ^58^ ^]^ The selection of components for constructing MPNs is vast, encompassing a broad range of polyphenols with a catechol structure as well as numerous metal ions that have been documented to possess the capability of building MPNs (Figure 3b).

The MPN boasts an array of functions and features, with the following four pivotal areas standing out as critical highlights. I) Synergization: MPNs combine the bioactivities of polyphenols and metal ions to achieve synergistic therapeutic effects. For example, Hong et al. constructed nanosheet MPNs composed of epigallocatechin‐3‐gallate (EGCG) and Cu^2+^, combining the antioxidant effect of EGCG and the anti‐inflammatory effect of Cu^2+^. Cu‐EGCG nanosheets act as scavengers of ROS, promote the transformation of macrophages to the M2 phenotype, and exerted anti‐inflammatory potential against osteoarthritis (Figure 3c).^[^ ^61^ ^]^ II) Drug delivery and in the form of coatings: On the one hand, MPNs are able to form nanoparticle products as drug delivery carriers with good carrying capacity for a variety of functional molecules such as horseradish peroxidase, glucose oxidase, bovine serum albumin and insulin.^[^ ^62^ ^]^ Moreover, MPNs are capable of forming thin films on the surfaces of hydrophobic drugs,^[^ ^63^ ^]^ peptide coacervates,^[^ ^64^ ^]^ hollow mesoporous nanoparticles,^[^ ^65^ ^]^ hydroxyapatite,^[^ ^66^ ^]^ polyamide membrane,^[^ ^54^ ^]^ polydopamine,^[^ ^67^ ^]^ cardiovascular stents,^[^ ^68^ ^]^ cells,^[^ ^69^ ^]^ tissues,^[^ ^70^ ^]^ etc., which can bring the bioactivities possessed by MPNs to these substances without affecting their properties (Figure 3d). III) Stimulus responsiveness: MPNs are stimulus‐responsive to three factors: pH, glutathione (GSH), and adenosine triphosphate (ATP). Thus, they can achieve responsive separation and release in specific situations. pH‐responsiveness is achieved by affecting the protonation of the phenolic hydroxyl groups of polyphenols. Distinctly, both GSH and ATP achieve release of MPNs by competitive coordination with metal ions and thus release of MPNs.^[^ ^71^ ^]^ For example, GSH competes with tannic acid for coordination with Fe^3+^ in the study of Sanghoon Kim et al: at pH 7, log K = 11 for GSH and Fe^3+^ complexes and log K = 9 for tannic acid and Fe^3+^ complexes, so GSH is more competitive at this pH, leading to the release of MPNs (Figure 3e).^[^ ^72^ ^]^ IV) Catalytic ability: The electrochemical properties between polyphenols and metal ions make the MPNs an electrochemically active system with initial oxidative reactivity derived from the quasi‐reversible reduction of metal ions and the irreversible oxidation of ligands.^[^ ^73^ ^]^   In many research strategies, such electrochemical properties can be well combined with the catalytic properties of metal ions. Most characteristically, both Fe^3+^ and Fe^2+^ are catalytic for Fenton and Fenton‐like reactions (reactions that convert hydrogen peroxide to reactive oxygen species), but Fe^3+^ in the oxidized state is less catalytic than Fe^2+^ in the reduced state. For the MPNs system, polyphenols happen to act as a reducing agent from Fe^3+^ to Fe^2+^. In short, this leads to the last point that MPNs can facilitate the conversion of metal ions between oxidized and reduced states by utilizing the reducibility of polyphenols. This process enhances the catalytic ability of metal ions, promoting Fenton reactions and ultimately generating ROS, which can be beneficial in disease treatment. For example, Zhang et al. reported how tannins in the tumor environment reduce Fe^3+^ to Fe^2+^, catalyzing the generation of ROS from hydrogen peroxide to inhibit tumors. Moreover, Fe^2+^ will be oxidized in the process, forming a cycle that utilizes Fe^2+^ and Fe^3+^.^[^ ^71^ ^]^ Furthermore, it has been reported that endogenous substances can also reduce Fe^3+^. For instance, Feng et al. used Fe^3+^ and shikonin (a naphthoquinone) coordination to form nanomedicines against tumors via ferroptosis therapy. This was achieved by intracellular GSH reduction of Fe^3+^ to Fe^2+^, which stimulated the Fenton reaction and generation of ROS (Figure 3f).^[^ ^74^ ^]^

##### Properties Derived from the Structure of Catechol or Pyrocatechol

The following three distinctive features, stemming inherently from the structure of catechol or pyrocatechol, are concisely summarized and emphasized to demonstrate the potential properties that materials crafted with the integration of polyphenols featuring the catechol or pyrocatechol‐structure may exhibit. I) PH‐responsiveness: The effect of pH on catechol or pyrogallol is through influencing deprotonation and protonation of phenolic hydroxyl groups. This allows the MPNs^[^ ^75^ ^]^ and BPNs constructed through polyphenols with catechol or pyrogallol structure to dissociate under the influence of pH, transforming from a multiple complex (double or triple complex) to a single complex, thus releasing the encapsulated drug or the constructed structure (polyphenol, metal ions, phenylboronic acid) itself. Such specific pH environments include gastric acidic environments, low pH wound microenvironments,^[^ ^76^ ^]^ acidic tumor microenvironments^[^ ^77^ ^],^ and so on. Shen et al. used Fe^3+^ and quercetin to self‐assemble into MPNs shells for oral delivery. These shells encase a core of ionic liquids that enhance medication solubility and tissue permeability. The MPN shell is structurally stable in physiological circumstances and enables targeted medication release in the stomach following oral delivery.^[^ ^60^ ^]^ In the study by Li et al., Sm^3+^ and EGCG were assembled into spherical nanoparticles released in the acidic environment of tumors. The nanoparticles can inhibit melanoma by activating caspase‐3/7 and poly ADP ribose polymerase.^[^ ^73^ ^]^ II) Bioadhesion ability: Materials assembled with the participation of polyphenols have an adhesive capacity derived from the catechol or pyrogallol's abilityto readily interact with adhered surfaces. Among the polyphenolic compounds, tannins have a strong adherence capacity, which is related to higher number of catechol structures present in them. Therefore, various thin films and coatings based on MPNs have been extensively investigated, and they are not only of controllable thickness, but also capable of adhering to the surfaces of a diverse range of materials such as variety of inorganic materials, pharmaceuticals, living cells and tissues.^[^ ^78^ ^]^ Furthermore, applying polyphenol coatings or films to the surface of a material can harness the bioactivity of polyphenols and therefore increase the biological properties of the material. Shi et al. reported that they used rare‐earth ions and catechin self‐assembly to form a nanocoating on the surface of polyamide membranes, which prevented bacterial colonization (Figure 2d).^[^ ^54^ ^]^ The metal‐phenol coating formed by assembling Cu^2+^, and tannic acid had anti‐inflammatory and antibacterial effects in the study by Li et al.^[^ ^79^ ^]^ III) Antioxidant capacity: Polyphenols may function as antioxidants because of their many phenolic hydroxyl groups that possess reducing capabilities, and the quantity and arrangement of these groups impact their reducing ability.^[^ ^80^ ^]^ In short, polyphenols can achieve the elimination of free radicals (e.g., hydroxyl radical HO·) by transferring hydrogen atoms from the active OH group (s) of polyphenols to free radicals (Ar‐OH+R·→ ArO·+ RH).^[^ ^51^ ^]^ In addition to reducing free radicals, polyphenols can further reduce metal ions. Polyphenols will be oxidized by some metal ions such as Au^3+^, Ag^+^, Pd^2+^, and Pt^2+^ to quinone or semi‐quinone, while the metal ions are reduced to their metallic forms after gaining electrons from quinone.^[^ ^58^ ^]^ For example, Yang et al. prepared silver (Ag) nanoparticles on the surface of selenium (Se) nanowires, which was achieved by the reduction of Ag^+^ by EGCG with antibacterial^[^ ^81^ ^]^ and antioxidant^[^ ^82^ ^]^ effects, then resulting in the co‐assembly of Se@Ag@EGCG nanoparticles with synergistic antibacterial effects.^[^ ^83^ ^]^ However, it is important to note that not all polyphenols are antioxidants. Some polyphenols (e.g., monosubstituted phenols) may act as pro‐oxidants through auto‐oxidation, generating semiquinone and superoxide (O2·^−^) radicals that will damage DNA and proteins.^[^ ^84^ ^]^

In summary, this review outlines three strategies for the self‐assembly systems of polyphenols possessing a catecholamine structure: direct self‐assembly or co‐assembly with other NPHMs, assembly with phenylboronic acid to BPNs, and coordination with metal ions to form MPNs. It is noteworthy that this unique structure confers some outstanding characteristics to materials assembled with polyphenols having this particular structure: pH‐responsiveness, bioadhesive properties, and antioxidant capabilities. Future self‐assembly investigations have the flexibility to tailor these strategies based on specific application requirements.

#### 3.1.2 Self‐assembly of Other Polyphenols

##### Self‐assembly of Curcumin

Curcumin, a primary active compound found in Curcuma spp, is classified under phenolic acids within the polyphenols group. It exhibits various biological activities such as anti‐inflammatory, anti‐viral, anti‐cancer, anti‐amyloid, and antioxidant properties.^[^ ^85^ ^]^ Nevertheless, curcumin has limited water solubility and low bioavailability.^[^ ^86^ ^]^ The key structure of curcumin is a bis‐α, β‐unsaturated β‐diketones with keto‐enol tautomerism. At pH 3–7, the bis‐keto form is predominant, while at pH >8, the enol isomer dominates.^[^ ^87^ ^]^ Curcumin alone has not been found to self‐assemble directly; rather, it requires the facilitated co‐assembly of other molecules to produce supramolecular materials. The bis‐α, β‐unsaturated β‐diketones structure is crucial for this co‐assembly process. A typical example is the self‐assembly of monosaccharides such as glucose and fructose with curcumin in aqueous solution to form spherical hollow capsules through hydrogen bonding facilitated by the bis‐α, β‐unsaturated β‐diketones structure of curcumin.^[^ ^88^ ^]^

In addition, metal ions are worth focusing on in the selection of substances that promote curcumin. For instance, in the triple co‐assembly of 5‐aminolevulinic acid, Fe^3+^, and curcumin to form nanodrug for continuous spatiotemporal therapy against tumors reported by Fang et al, the formation of the product mainly relied on the interactions formed between Fe^3+^ and curcumin to play a dominant role.^[^ ^89^ ^]^ Metal coordination interactions between metal ions and curcumin also help stabilize the bis‐α, β‐unsaturated β‐diketones structure, preventing its destabilizing changes. Li et al. prepared amino acid coordination‐driven self‐assembled curcumin nanoparticles using curcumin, amino acids, and Zn^2^^+^, effectively alleviating the problems of rapid curcumin degradation and poor tissue uptake, and enhancing curcumin accumulation in tumor tissues.^[^ ^90^ ^]^

In conclusion, for the self‐assembly process of curcumin, we emphasize the importance of fully utilizing its bis‐α, β‐unsaturated β‐diketone structure to promote self‐assembly through interactions such as metal coordination and hydrogen bonding.

##### Self‐assembly of Honokiol and Others

Lignans are polyphenols formed by the oxidative polymerization of several (usually two) phenylacetones (phenylacetone consisting of a benzene ring and three straight‐chain carbons with the structural unit C6‐C3). Honokiol, extracted from the bark of the traditional Chinese herb Magnolia officinalis, is one of the representative natural polyphenolic small molecules in the lignans class, with rich biological activities such as anti‐advanced nonsmall cell lung cancer, anti‐viral, and improvement of pulmonary fibrosis.^[^ ^91^ ^]^ The formation of hydroxyl groups and *π*–*π* stacking interactions allow lignans to self‐assemble into nanoparticles (HK‐NPs), according to Ji et al. HK‐NPs are subsequently endocytosed by tumor cells and disassembled in lysosomes, releasing hydroxyl groups into the cytoplasm and nucleus. This process ultimately leads to the degradation of the mutp53/TBK1 complex.^[^ ^92^ ^]^ In addition, other natural polyphenolic small molecule self‐assemblies include resveratrol self‐assembled with Nnicotinamide riboside^[^ ^93^ ^]^ and piperine,^[^ ^94^ ^]^ respectively; cinnamic acid self‐assembled with berberine^[^ ^95^ ^]^ constituting a dual natural small molecule self‐assembly system.

### 3.2 Self‐assembly of Quinones

Quinones constitute a class of NPHMs that possess an unsaturated cyclic diketone structure, exhibiting a wide array of bioactivities such as anti‐tumor,^[^ ^96^ ^]^ anti‐bacterial,^[^ ^97^ ^]^ anti‐viral,^[^ ^98^ ^]^ and so on, making them valuable in drug development.^[^ ^99^ ^]^ The self‐assembly strategies for quinones encompass the formation of metal‐quinone networks through metal coordination, as well as the self‐assembly of quinones alone or in conjunction with other NPHMs. This section initially delves into the structural characteristics and self‐assembly mechanisms of quinones, subsequently exploring the metal‐quinone network and the self‐assembly process with other NPHMs.

#### 3.2.1 Structure Commonality Related to Self‐Assembly Mechanism

**Figure 4:**
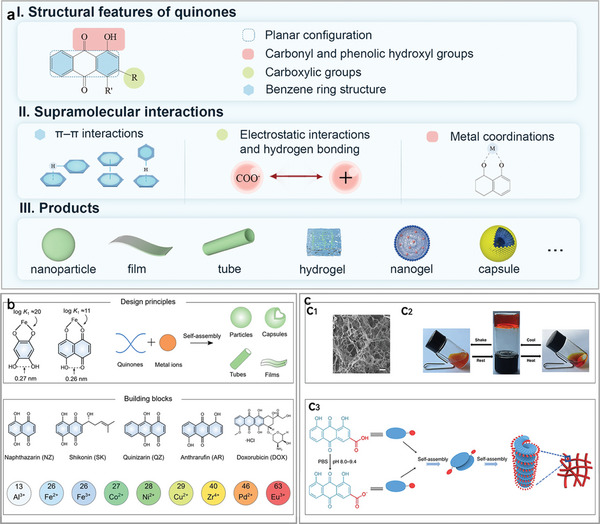
a) Schematic representation of the quinone self‐assembly mechanism: from key structures affecting self‐assembly, key drivers, to supramolecular materials. b) Assembly principle of the metal‐quinone network showing the distances of neighboring oxygen atoms in the catechol and quinone molecules and the different stabilization constants for coordination with Fe^3+^. Alternative building blocks offer insights into the structural formulations of select quinones and metal ion species. Reproduced with permission. Reproduced with permission.^[^ ^100^ ^]^ Copyright 2023, Wiley. C_1_) SEM image of the rhein self‐assembled hydrogel. C_2_) Shear stress and temperature‐induced gel‐sol reversible transition in rhein hydrogels. C_3_) Self‐assembly steps of rhein hydrogels: nanofibers in left‐handed helical configuration were first self‐assembled by *π*–*π* stacking, hydrogel bonding and electrostatic interactions, and then further cross‐linked into hydrogels. Reproduced with permission.^[^ ^11^ ^]^ Copyright 2019 Springer Nature.

Quinones exhibit distinct structural features that are intimately related to their self‐assembly mechanisms and strategies. I) Planar configuration. II) Carbonyl and phenolic hydroxyl groups: Some quinones have adjacent oxygen atoms, thus providing sites capable of coordinating with metals, such as those containing 1,8‐naphthoquinone moieties.^[^ ^100^ ^]^ In addition, these structures can also promote self‐assembly by hydrogen bonds. III) Carboxylic groups: electrostatic interactions and hydrogen bonding produced by the carboxylic groups are essential for the self‐assembly of certain quinones, such as rhein.^[^ ^11^ ^]^ IV). Benzene ring structure: *π*–*π* interaction between quinone benzene rings is an important driver for their self‐assembly.^[^ ^101^ ^]^ Based on the above structural and self‐assembly features, the self‐assembled form of quinone will be summarized in the next section (**Figure** **4**a).

#### 3.2.2 Self‐Assembly of Quinones with Mental Ions: Metal‐Quinone Networks

The carbonyl and phenolic hydroxyl groups of quinones act as coordination sites, facilitating the creation of a metal‐quinone network.^[^ ^102^ ^]^ To create metal quinone networks, Dr. Qizhi Zhong and colleagues used quinones with 1,8‐naphthoquinone fractions. This material has the following characteristics: I) A variety of building components are found in quinone fractions: quinone fractions include naphthazarin, shikonin, quinizarin, anthrarufin, doxorubicin (DOX) and so on. Al^3+^, Fe^2+^, Fe^3+^, Co^2+^, Ni^2+^, Cu^2+^, Zr^4+^, Pd^2+^, and Eu^3+^ are examples of liganding metals. II) The product has a diverse form, being able to autonomously assemble into particles, tubes, capsules, films, and several other materials. III) The metal‐quinone network exhibits distinct coordination bond strengths when compared to the MPNs. For example, the stability constant log K is ≈11 for Fe^3+^ binding to naphthoquinone, which is lower than log K of ≈20 for Fe^3+^ binding to the polyphenol catechol. IV). The metal‐quinone network exhibits dual pH‐responsiveness, enabling decomposition and release under both acidic and alkaline conditions, hence facilitating multi‐responsive drug release. V). The quinones and metal ions, which are the building blocks, possess various biological activities. Consequently, the self‐assembled products have therapeutic effects without requiring additional medications (Figure 4b).^[^ ^100^ ^]^ Feng et al. directly self‐assembled nanoparticles using the coordination of Fe^3+^ and shikonin at HO─C and C═O sites, and the product could exhibit anti‐tumor activity by inducing ferroptosis.^[^ ^74^ ^]^ In conclusion, compared to MPNs, which have been widely studied and applied, the study of metal‐ quinone networks is still in its early stages, and more research and biological basis are needed to confirm their roles.

#### 3.2.3 Other Quinones Self‐Assembly Products

Rhein is an NPHM that exerts therapeutic advantages in a variety of diseases, such as ischaemic stroke,^[^ ^103^ ^]^ ulcerative colitis,^[^ ^104^ ^]^ antibacterial,^[^ ^105^ ^]^ and heart failure.^[^ ^106^ ^]^ A landmark achievement was the discovery by Zheng et al. that rhein can act as an NPHM hydrogel agent to self‐assemble to form supramolecular hydrogels (Figure 4c). For the self‐assembly process of rhein, we need to pay attention to the fact that the carboxyl group and planar conformation of rhein are closely related to its self‐assembly properties. An experimental phenomenon we can observe is that pH significantly affects the hydrogelation of rhein. This is due to the different situations of protonation and deprotonation of the carboxyl groups at different pH values, which in turn affects the supramolecular self‐assembly driven by hydrogen bonding and electrostatic interactions. Thus, only in the pH range of 8.0 to 9.4, both carboxylate deprotonated sodium salt of rhein and carboxylate unprotonated monomer of rhein exist, where there is electrostatic repulsion between carboxylate ions and electrostatic mutual attraction between carboxylate and carboxylate ions. At the same time, the planar conformation of rhein allows it to organize itself in opposite directions, with parallel stacking between planes, thus self‐assembling into dimers via J‐polymerization. The dimer is eventually converted into a hydrogel with a 3D structure. For the formation of rhein hydrogels, which are not only thermally reversible, self‐healing and injectable, but can also bind to the active site of toll‐like receptor 4 and demonstrate anti‐neuroinflammatory effects by inhibiting the TLR4/NFκB signaling pathway.^[^ ^11^ ^]^ Subsequently, Yun Hao et al. undertook a research to investigate the process and results of pH‐induced self‐assembly of rhein by dissipative particle dynamics (DPD) simulation. Results showed that rhein formed insoluble precipitates in acidic conditions, mostly appearing as spherical, columnar, and membrane‐like structures. In an alkaline environment, the self‐assembled products showed improved water solubility and had a structure resembling spheres and worms. Furthermore, the formation and structure of the 3D network were influenced by the extent of carboxyl deprotonation.^[^ ^107^ ^]^ The results once again indicate a relationship between the self‐assembly of rhein and its anthraquinone ring and carboxyl group. The applications of rhein hydrogels in novel oral therapeutics have been further developed in subsequent studies. According to Zhong et al. the rhein hydrogel can be loaded with Spirulina platensis, which achieves sustained rhein release in a simulated intestinal environment and plays an important role in inflammatory bowel disease as well as psychological disorders associated with inflammatory bowel disease.^[^ ^108^ ^]^ In addition, quinones can be co‐assembled with other substances, especially alkaloid NPHMs such as berberine, coptisine,^[^ ^101^ ^]^ DOX, etc., to form a series of products such as nanogels, nanoparticles, nanofibers, nanogels, etc., which can play a therapeutic role in antimicrobial,^[^ ^109^ ^]^ anti‐ metastatic breast cancer,^[^ ^110^ ^]^ anti‐hepatoma by targeting mitochondria,^[^ ^111^ ^]^ and anti‐Alzheimer's diseases by inhibiting cholinesterase, modulating β‐amyloid and eliminating ROS.^[^ ^112^ ^]^

### 3.3 Self‐assembly of Monosaccharides

Carbohydrates are a crucial energy source for the human body and play a significant role in in various physiological processes such as intercellular recognition, protein transport,^[^ ^113^ ^]^ and have potential for use in supramolecular chemical self‐assembly because of their polyhydroxyl groups.^[^ ^114^ ^]^ Monosaccharides are the basic components of carbohydrates, which usually refer to polyhydroxy aldehydes or polyhydroxy ketones that cannot be further hydrolyzed. Research on monosaccharide self‐assembly has primarily concentrated on designing and synthesizing sugar‐based amphiphiles. These molecules typically consist of hydrophobic chains, such as alkyl chains, attached to hydrophilic monosaccharide heads.^[^ ^115^ ^]^ For example, galactose‐ and glucose‐based amphiphiles can self‐assemble into spherical micelles.^[^ ^116^ ^]^ In addition, very few studies have noted that monosaccharides can self‐assemble as independent individuals without structural modification, a process closely related to hydrogen bonding formed by monosaccharide hydroxyl groups. Sandy Wong et al. reported that the self‐assembly of monosaccharides and curcumin via hydrogen bonding into spherical capsules of 100–150 nm in size could be achieved by simply adding the monosaccharides and curcumin to water.^[^ ^88^ ^]^ However, they observed that glucose and fructose were able to perform this process, whereas mannose and galactose were not and only formed aggregates and precipitates. The strength of hydrogen bonding interactions between carbohydrate hydroxyl groups is related to the relative configuration of the hydrogen bonds in the following order: axial–axial > axial–equatorial > equatorial–equatoria.^[^ ^117^ ^]^ This could provide a referable explanation for Sandy Wong et al.’s observation that mannose and galactose, which have a higher proportion of axial ‐OH groups, are more inclined to form intramolecular rather than intermolecular hydrogen bonds, and therefore they do not exhibit co‐assembly. Taking this a step further, the team went on to explore the ability of the co‐assembled products of monosaccharides and curcumin to encapsulate drugs. Furthermore, the researchers investigated the capacity of the co‐assembled products of monosaccharides and curcumin to encapsulate pharmaceuticals. All hydrophobic drugs like albendazole, paclitaxel, and sulfasalazine were successfully encapsulated, except for warfarin. On the other hand, drugs more hydrophilic than curcumin such as gemcitabine and DOX could not be encapsulated due to their potential to disrupt the interaction between curcumin and fructose, leading to carrier instability. However, the biological effects of the carriers are subject to further experiments.^[^ ^118^ ^]^ In conclusion, the self‐assembling systems that monosaccharides are involved in constructing are of interest because they are a simple, readily available, and essential energy substance for human beings to sustain life activities on a daily basis. Therefore, their self‐assembly capacity and the potential applications of self‐assembled carriers they are involved in constructing deserve more attention.

### 3.4 Self‐assembly of Saponins

Saponins are a class of glycosidic NPHM that are widely distributed in over 500 species of plants and some marine organisms, such as starfish and sea cucumbers.^[^ ^119^ ^]^ Numerous research has highlighted their diverse biological properties, including antiviral, anti‐inflammatory, antitumor, and hypoglycemic effects.^[^ ^120^ ^]^

#### 3.4.1 Structure Commonality Related to Self‐Assembly Mechanism

**Figure 5:**
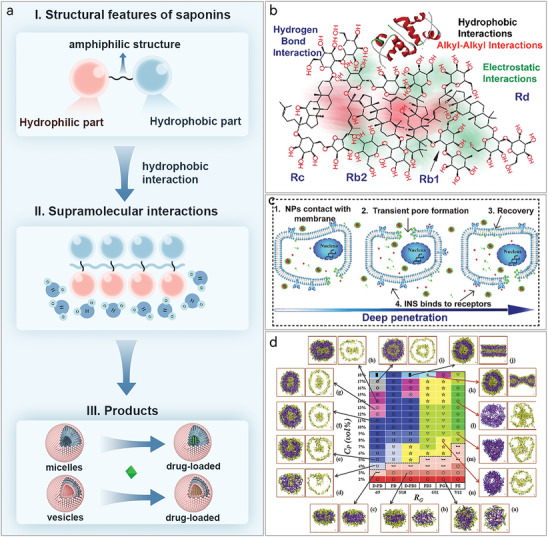
a) Schematic representation of saponin self‐assembly: saponins characterized by amphiphilic structures self‐assemble under hydrophobic interaction to form supramolecular materials represented by micelles and vesicles. b) Supramolecular interaction forces that mimic the self‐assembly of insulin (INS) and ginsenosides (Rd, Rc, Rb1, Rb2), including hydrogen bond, hydrophobic interaction, alkyl‐alkyl interaction and electrostatic interaction. Reproduced with permission.^[^ ^123a^ ^]^ Copyright 2021, Elsevier. c) Schematic diagram of ginsenoside self‐assembled nanoparticles (NPs) carrying insulin to permeate the cell membrane. (1) NPs in contact with cell membrane. (2) NPs interact with the cell membrane by binding to cholesterol, forming transient pores that allow NPs to enter the cell. (3) Membrane integrity is restored. Reproduced with permission.^[^ ^123a^ ^]^ Copyright 2021, Elsevier. d) Morphological maps of saponin self‐assembly products in terms of ratio of the hydrophilic structures (R_G_) and the saponins’ concentration (C_P_) simulated by dissipative dynamics. a) spherical micelles, b) ellipse micelles, c) oblate micelles, d,e) semivesicles, f) spherical vesicles, g) ellipse vesicles, h) oblate vesicles, i) multilamellar vesicles, j) tubules, k) necklace‐like micelle, l) basket‐shaped multicompartment vesicles, m) tee shaped multicompartment vesicles, n) tetrahedron shaped multicompartment vesicles. Reproduced with permission.^[^ ^132^ ^]^ Copyright 2015, Elsevier.

Synthesizing amphiphilic molecules to produce supramolecular products is an effective approach in self‐assembly material synthesis.^[^ ^121^ ^]^ However, saponins, a natural amphiphilic molecule gifted by nature, can self‐assemble without the need for artificial synthesis or structural modification, making them particularly noteworthy. A representative structural feature influencing saponin self‐assembly is that saponins consist of two parts: a hydrophilic part consisting of one or more sugar chains and a hydrophobic part consisting of triterpenes (mainly pentacyclic triterpenes or tetracyclic triterpenes) or spirostane.^[^ ^119^ ^]^ Under hydrophobic interactions, the hydrophilic and hydrophobic portions of saponins are brought closer together, with the hydrophilic portion oriented toward the polar solution environment and the hydrophobic portion oriented away from it. This arrangement ultimately leads to the self‐assembly of saponins into micelles and vesicular supramolecular products with hydrophobic hollow structures, forming a type of nanoparticle (**Figure** **5**a). Saponins have gradually become a prominent focus of self‐assembly studies in NPHM in recent years. Next, we will summarize the characteristics of different types of saponin self‐assembly products.

#### 3.4.2 Saponins Self‐Assembly Products: Micelles and Vesicles

Micelles are the primary self‐assembled structures formed because of  the amphiphilic properties of saponins. Hydrophobic interactions are the primary driving force behind this process. When the concentration surpasses the critical micelle concentration (CMC), a micellar structure is formed with a hydrophilic outer layer and a hydrophobic inner layer.^[^ ^122^ ^]^

The potential of saponin self‐assembled micelles as drug delivery carriers warrants attention and will be discussed in the following areas. I) As efficient vehicles for delivering hydrophobic drugs: saponin micelles may encapsulate hydrophobic pharmaceuticals in their hydrophobic core, shielded by the hydrophilic outer layer, therefore improving the water solubility of these medications.^[^ ^123^ ^]^ Encapsulation of the drug also supports self‐assembly and stability.^[^ ^124^ ^]^ For example, Quillaja saponins in aqueous solution can self‐assembly into micelles with a diameter of 7.5 mm and can load lutein esters,^[^ ^125^ ^]^ and ginsenoside Rg1, Re, Rf, Rb1, Rb1, Rc, Rb2, Rb3, Rd,^[^ ^123^, ^126^ ^]^ can be self‐assembled into nanoparticles for delivery of insulin via hydrogen bond, hydrophobic interaction, alkyl‐alkyl interaction and electrostatic interaction (Figure 5b). GA,^[^ ^12^, ^18^, ^124^, ^127^ ^]^ etc., can be self‐assembled into micelles for delivery of tanshinone IIA,^[^ ^10^ ^]^ DOX,^[^ ^123b^ ^]^ curcumin,^[^ ^128^ ^]^ paclitaxel,^[^ ^129^ ^]^ and many other hydrophobic drugs. II) Enhancing cell entrance and penetration: Enhancing cellular entry and penetration: Saponin self‐assembled micelles can facilitate the entry of encapsulated medicines into cells. For instance, ginsenoside self‐assembled micelles containing insulin may interact with the cell membrane by binding to cholesterol, resulting in the creation of a transient pore. The insulin‐encapsulated ginsenoside carrier then passes through, after which the membrane regains its integrity (Figure 5c).^[^ ^123a^ ^]^ III) Synergistic effect: natural saponins have biological characteristics including anticancer, anti‐inflammatory, and so on,^[^ ^130^ ^]^ enabling them to serve as drug delivery carriers while also providing synergistic therapeutic effects with the delivered drug. For example, delivering levofloxacin using micelles formed by the self‐assembly of 3‐O‐β‐D‐Glucopyranosyl platycodigenin 682, a saponin NPHM, can promote the enrichment of levofloxacin in lung tissues, thereby enhancing its anti‐infective effect.^[^ ^131^ ^]^

In addition to the supramolecular form of micelles, Xing et al. used DPD to show that saponins have the potential to form a self‐assembled products in a variety of forms. What influences the form of the product? The results showed that the higher the proportion of the saponin sugar (hydrophilic portion), the more compartments were contained in the vesicles formed. Hence, the proportion of the hydrophilic part (sugar chain) to the hydrophobic part of the saponin may significantly impact the varied shapes of the self‐assembled products, such as whether they develop vesicular or porous formations (Figure 5d).^[^ ^132^ ^]^ There is also evidence of a correlation between the diameter of the product and the dose ratio of the encapsulated drug to saponin.^[^ ^123a^ ^]^ Therefore, many factors need to be considered when preparing saponin self‐assembled products, including the ratio of the amphiphilic structure of the saponin and the dose ratio of the encapsulated drug to saponin, among others.

In conclusion, saponins are representative of NPHMs with amphiphilic structural features. Their supramolecular micelles are regarded as a promising nanoparticle, especially for their ability to synergistically exert drug‐delivering capacity and many pharmacological effects, which deserves the potential of further research.

#### 3.4.3 Saponins Self‐Assembly Products: Hydrogels and Others

GA, a saponin extracted from licorice root,^[^ ^133^ ^]^ exhibits various biological activities, including anti‐ inflammatory,^[^ ^134^ ^]^ anti‐viral,^[^ ^135^ ^]^ anti‐tumor,^[^ ^136^ ^]^ and so on. It is capable of directly self‐assembling to form a hydrogel, making it one of the most representative NPHM‐based supramolecular bioactive hydrogels. The next section will present GA as a representative example, focusing on its structure and self‐assembly strategy.

**Figure 6:**
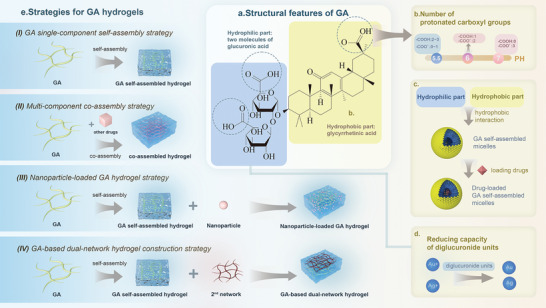
A comprehensive mind map of glycyrrhizic acid (GA) from structure to self‐assembly strategies. a) Molecular formula of the amphiphilic structure of GA, with the yellow portion indicating the cooked portion and the blue portion indicating the hydrophilic portion. b) Effect of pH on the structure of GA. c) Self‐assembly of GA with amphiphilic structure into drug‐loadable micelles. d) Reducing ability of hydrophilic portion of GA. e) Four major strategies for self‐assembly of hydrogels based on GA.

Structurally, GA has the following characteristics: I) GA, belonging to the saponin family, also exhibits a structural composition marked by two amphiphilic components. Its hydrophobic part comprises a single molecule of pentacyclic triterpene 18β‐ glycyrrhetinic acid, while the hydrophilic segment consists of  two molecules of glucuronic acid (**Figure** **6**a).^[^ ^137^ ^]^ This inherent amphiphilicity inherently equips GA with the basic ability to self‐assemble into micelles and vesicles, driven by hydrophobic forces. (Figure 6c). II) The GA molecule features three carboxyl groups, two located on glucuronic acid and one on a triterpene. The pH level significantly influences the ionization of these carboxyl groups, subsequently altering the formation of hydrogen bonds, self‐assembly processes, as well as the development of micelles and gel networks. At pH values less than or equal to 5, the carboxyl group of the triterpene fragment and at least one of the two carboxyl groups (pK = 4.4, 5.3) of the glucuronic fragment become protonated. However, at pH 6, solely the carboxyl group of the triterpene fragment undergoes protonation (pK = 6.9).^[^ ^138^ ^]^ When pH > 7, only GA monomers were present.^[^ ^139^ ^]^ The critical pH range for achieving a balance between the hydrophilic and hydrophobic properties of GA is 5–8, with micelles specifically forming within the narrower pH range of 5–6 (Figure 6b).^[^ ^139^ ^]^ III) The diglucuronic acid unit of GA exhibits reductive properties, a valuable characteristic that can be harnessed in the development of materials. For example, this unit possesses the ability to spontaneously reduce heavy metal ions in situ, converting them into metal nanoparticles like gold nanoparticles,^[^ ^140^ ^]^ and silver nanoparticles.^[^ ^141^ ^]^ (Figure 6d).

The self‐assembly products of GA mainly include micelles, nanofibers with right‐handed helices, and hydrogels.^[^ ^140b^ ^]^ Saponin‐based micelles were introduced previously, and this section will focus on the hydrogel form. This review broadly categorizes the strategies for GA self‐assembly studies into the following categories (Figure 6e). I) GA single‐component self‐assembly strategy: Initial research focused exclusively on investigating the characteristics and potential uses of self‐assembled hydrogels composed solely of GA, which exhibit injectability, antibacterial properties, anti‐inflammatory effects, and the ability to facilitate wound healing.^[^ ^142^ ^]^ Nonetheless, the hydrogels exhibited inadequate mechanical strength and adhesion properties. II) Multi‐component co‐assembly strategy: This strategy aims to utilize GA and other NPHM or other functional molecules to construct a multi‐component co‐assembly system, with each component exerting a specific therapeutic effect, and ultimately producing a synergistic effect. For example, Wenmin Pi et al. reported a three‐component co‐assembled hydrogel with GA, copper ions and norcantharidin, which can synergistically inhibit tumor proliferation, invasion and metastasis in three aspects: apoptosis, cuproptosis and anti‐inflammation.^[^ ^16^ ^]^ In addition, the co‐assembly of metal ions and GA can promote gel formation. Metal ions can enhance gel formation, with the effectiveness observed in the order of Zn^2^⁺ > Cu^2^⁺ > Ca^2^⁺.^[^ ^143^ ^]^ III) Nanoparticle‐loaded GA hydrogel strategy: GA hydrogels can be well loaded with nanoparticles, such as metal nanoparticles,^[^ ^140a^ ^]^ graphene oxide,^[^ ^140b^ ^]^ ferrous sulfide nanoparticles,^[^ ^144^ ^]^ protein–polysaccharide complex nanoparticles,^[^ ^145^ ^]^ carbon quantum dots,^[^ ^146^ ^]^ etc. GA hydrogels provide loaded nanoparticles with slow‐release effects,^[^ ^146^ ^]^ synergistic bioaugmentation (e.g., enhanced antimicrobial properties^[^ ^146^ ^]^), and the ability to reduce metal ions to metal nanoparticles,^[^ ^140a^ ^]^ among others. In addition, the loaded nanoparticles bring different functions to the GA‐based hydrogel system, such as catalysis.^[^ ^140b^ ^]^ (IV) GA‐based dual‐network hydrogel construction strategy: The construction of GA‐based dual‐network hydrogel system has been reported, including the introduction of a second gel network^[^ ^140^, ^147^ ^]^ that enhanced the mechanical strength.^[^ ^148^ ^]^ In the GA‐based dual‐network hydrogel system, the GA hydrogel primarily provides strong bioactivity, while the second layer network formed by polymers contribute high mechanical strength and adhesion. These two are complementary, enhancing the overall performance of the system.

To summarize, based on the above strategies, we suggest the following three points in the hope of inspiring future research.

Firstly, it is crucial to recognize the focus and advantages of different self‐assembly strategies. For example, the advantage of a multicomponent co‐assembled supramolecular hydrogel is that it is composed of multiple NPHM, which can exert their respective therapeutic efficacies at the designated site, achieving multi‐pathways synergistic effects. In the construction of nanoparticle‐carrying hydrogels, intravenously injected nanoparticles can be delivered and released in situ through the hydrogel, enabling local drug delivery and reducing systemic toxicity. For dual‐network supramolecular hydrogels, one major advantage is the improvement of mechanical properties. Therefore, future studies should select appropriate assembly strategies based on specific requirements. Secondly, different strategies are not mutually exclusive, and researchers should consider integrating them. Thirdly, researchers should not limit these strategies to GA hydrogels alone, as they are widely applicable to a range of supramolecular hydrogels. Experimenting with different NPHM to self‐assemble hydrogel substrates could achieve various therapeutic effects. Researchers should explore these possibilities to broaden the scope and efficacy of supramolecular hydrogels.

### 3.5 Self‐assembly of Alkaloids

Alkaloids are a class of nitrogen‐containing NPHM, and it is estimated that plants are capable of producing ≈12000 different alkaloids^[^ ^149^ ^]^ with a wide range of biological activities such as vasodilatory,^[^ ^150^ ^]^ neuroprotective,^[^ ^151^ ^]^ antibacterial,^[^ ^152^ ^]^ and antitumor^[^ ^153^ ^]^ activities. The current research on the self‐assembly of alkaloids in NPHMs is mostly centered on berberine. Due to the limited bioavailability of berberine,^[^ ^154^ ^]^ the self‐assembly process plays a crucial role in enhancing the use of berberine. Therefore, in this section, a systematic overview of the self‐assembly properties of berberine will be presented as a representative, in the expectation of providing insights into the self‐assembly of other alkaloids.

#### 3.5.1 Structural Commonality Related to Self‐Assembly Mechanism

**Figure 7:**
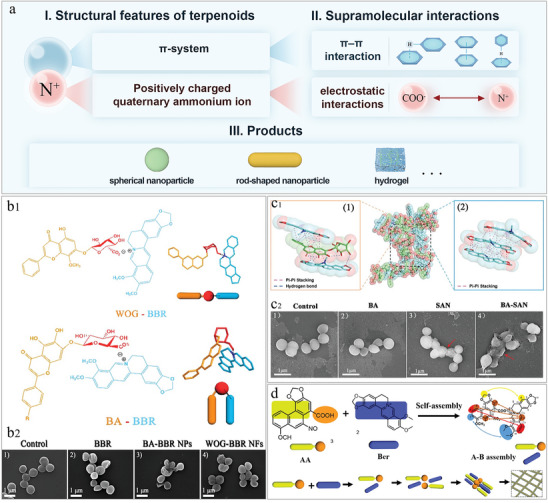
Self‐assembly of alkaloid. a) Schematic representation of berberine (BBR) self‐assembly strategy: from structural features, drivers to products. b_1_) BBR self‐assembled with baicalin (BA) and wogonoside (WOG), respectively, a process all driven by the electrostatic mutual attraction of the nitrogen positive ions of berberine. Reproduced with permission.^[^ ^9^ ^]^ Copyright 2019, ACS. b_2_) FESEM images of bacterial morphology. BBR was co‐assembled with BA and WOG into nanoparticles and nanofibers, respectively, with the former having better antimicrobial effect. Reproduced with permission.^[^ ^9^ ^]^ Copyright 2019, ACS. c_1_) Molecular interactions of sanguinarine (SAN) and BA co‐assembled into a hydrogel. hydrogen bonding and *π*–*π* stacking between sanguinarine and BA as shown in (1). *π*–*π* stacking between sanguinarine molecules as shown in (2). Reproduced with permission.^[^ ^156^ ^]^ Copyright 2023, Wiley. c_2_) FESEM images of bacterial morphology. The antimicrobial effect of hydrogels co‐assembled of BA and SAN is stronger than that of BA and SAN monomers. Reproduced with permission.^[^ ^156^ ^]^ Copyright 2023, Wiley. d) Schematic representation of the co‐assembly of berberine and aristolochic acid (AA) to form linear heterogenous supramolecules that hide the carboxyl site (orange sphere) of AA from being metabolized into toxic aristololactam. Reproduced with permission.^[^ ^17^ ^]^ Copyright 2021, ACS.

The following structural properties are suggested to be our focus for understanding the self‐assembly mechanisms and strategies involved in the alkaloids of NPHMs: I) Positively charged quaternary ammonium ion: positively charged quaternary ammonium ion is the most critical structure for the self‐assembly process. It is this because its positive charge can attract negatively charged groups close by electrostatic interactions, which is the most significant step in the self‐assembly of berberine with other molecules. **Table** **1** summarizes the substances that can self‐assemble with berberine, and it is easy to find that basically all of them have negatively charged groups, especially carboxyl groups. And in their progress of self‐assembly with berberine, the main driving force is the electrostatic interactions between the negatively charged carboxylic acid and hydroxyl groups and the positively charged quaternary ammonium ions of berberine. For example, in the self‐assembly of berberine with baicalin and flavonoid glycosides, respectively, the first step of the assembly relies on the formation of a 1D composite unit between the quaternary ammonium ions of berberine and the carboxylate ions of the flavonoids via electrostatic interactions (**Figure** **7**b_1_).^[^ ^9^ ^]^ During the self‐assembly of curcumin and berberine, curcumin is negatively charged due to the presence of phenolic hydroxyl groups, and positively charged berberine and negatively charged curcumin are attracted to each other with electrostatic attraction as the initial driving force.^[^ ^155^ ^]^ This suggests that when selecting molecules for self‐assembly with berberine, should researchers pay attention to those molecules that can generate negatively charged groups to ensure effective electrostatic interactions. II) π‐system: Berberine has a benzene ring structure, so *π*–*π* interactions also facilitate self‐assembly processes, such as in the self‐assembly of berberine with cinnamic acid^[^ ^95^ ^]^ and rhein.^[^ ^109^ ^]^ During the co‐assembly of sanguinarine, another alkaloid, and baicalin, *π*–*π* interactions drive the assembly between sanguinarine and between sanguinarine and baicalin (Figure 7c_1_).^[^ ^156^ ^]^ In conclusion, for ionic quaternary bases such as berberine, electrostatic interactions are a key step in self‐assembly as well as *π*–*π* interactions also promotes self‐assembly (Figure 7a). Among them, we should pay particular attention to the nitrogen atoms of alkaloids in the development of alkaloid self‐assembled nanomaterials. Based on the above structural and self‐assembly features, alkaloids can be self‐assembled into mainly nanoparticles and hydrogels, as described next.

**Table 1:** Laws of substances capable of co‐assembling with positively charged alkaloids.

Alkaloids (positively charged)	Structural formulae of alkaloids	Co‐assembled NPHMs (negatively charged)	Structural formulae of NPHMs	Products	References
berberine	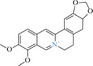	Baicalin	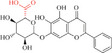	Nanoparticles	[9, 157]
		Rhein	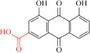	nanoparticles	[109]
		GA	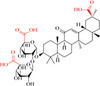	complex formulation	[158]
		Wogonoside	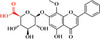	Nanofibers	[9, 157]
		aristolochic acid	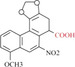	linear heterogeneous supramolecule	[17]
		cinnamic acid	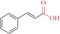	Nanoparticles	[95]
		3,4,5‐methoxycinnamic acid	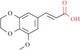	Nanoparticles	[159]
		hesprertin	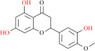	nanoparticles	[160]
		curcumin	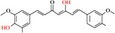	Submicron particles	[155]
sanguinarine	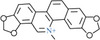	baicalin	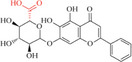	Hydrogel	[156]
coptisine	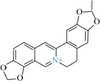	rhein	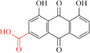	Nanofibers	[101]
		emodin	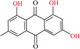	nanoparticles	[101]

#### 3.5.2 Alkaloids Self‐Assembly Products: Nanoparticles and Hydrogels

Alkaloids‐based supramolecular bioactive materials are mainly in the form of nanoparticles and hydrogels (Figure 7a), of which berberine‐based materials have two unique properties when fighting microorganisms: resistance and modulation. First, they are resistant to microorganisms that are harmful to humans. Supramolecular nanoparticles co‐assembled from berberine with a variety of NPHMs (e.g., rhein,^[^ ^109^ ^]^ cinnamic acid,^[^ ^95^ ^]^ curcumin,^[^ ^155^ ^]^ etc.) exhibited high bacterial inhibition, bacterial biofilm clearance, and were even stronger than first‐line antimicrobials such as norfloxacin, amoxicillin and tetracycline. Different forms of supramolecular materials of berberine lead to different strengths of antimicrobial effect. For example, Li et al. reported the co‐assembly of berberine with baicalin and wogonoside to form surface hydrophilic nanoparticles and surface hydrophobic nanofibers, respectively, in which the former hydrophilic nanoparticles had better antibacterial effects due to their stronger adhesion to bacteria (Figure 7b_2_).^[^ ^9^, ^161^ ^]^ In addition to nanoparticles, another alkaloid, sanguinarine, can be co‐assembled with baicalin to form supramolecular hydrogels with antimicrobial properties, a product form that can be applied as a dressing to infected wounds to promote their healing (Figure 7c_2_).^[^ ^156^ ^]^

On the other hand, in the face of intestinal flora, berberine‐based supramolecular materials can play a regulatory role. In the treatment of ulcerative colitis, diarrhea‐predominant irritable bowel syndrome, and other intestinal diseases, berberine‐based supramolecular nanoparticles exhibit modulation of gut microbiota. For example, co‐assembled nanoparticles of berberine and hesperetin can restore normal levels of *Erysipelotrichi, Bacilli, and Bacilli* in ulcerative colitis mice;^[^ ^160^ ^]^ co‐assembled nanoparticles of berberine and tannic acid can modulate the beneficial bacteria Lactobacillus gasseri and Lactobacillus murinu, resulting in a therapeutic effect on ulcerative colitis;^[^ ^162^ ^]^ for the treatment of diarrhea‐predominant irritable bowel syndrome, co‐assembled nanoparticles of berberine and baicalin were able to significantly reduce therelative abundances of *Verrucomicrobia*.^[^ ^157a^ ^]^ This means that it is promising to construct berberine‐based supramolecular material systems to treat intestinal diseases by targeting intestinal flora. In addition, for substances that disrupt the intestinal flora, the formation of berberine‐based supramolecular materials could also serve to protect that homeostasis. For example, the co‐assembly of aristolochic acid and berberine to form linear heterogenous supramolecules, which prevented the carboxyl group of structural aristolochic acid from being metabolized to toxic aristololactam through self‐assembly, significantly preventing the disruption of intestinal flora homeostasis by aristolochic acid (Figure 7d).^[^ ^17^ ^]^

### 3.6 Self‐assembly of Phytosterols

Phytosterols are found in all plants, with sitosterol, stigmasterol and campesterol being the three most common.^[^ ^163^ ^]^ The structure of phytosterols is similar to cholesterol, with cyclopentane polyhydrophenanthrene as the basic backbone. Hypolipidemic effects of phytosterols have been demonstrated, with their effects on cholesterol achieved by affecting cholesterol transport in the blood, regulating cholesterol metabolism in the liver, and inhibiting the absorption of cholesterol in the intestinal tract.^[^ ^164^ ^]^ Furthermore, phytosterols also has anti‐bacterial, anti‐ulcer, anti‐cancer, preventing non‐alcoholic fatty liver disease and many other biological activities.^[^ ^165^ ^]^ Nevertheless, three major challenges have been encountered in the application of phytosterols: limited water solubility, brief retention and inadequate absorption within the gastrointestinal tract, and susceptibility to degradation and cytotoxicity of the resulting products when exposed to light, heat, and oxygen.^[^ ^166^ ^]^ Based on these shortcomings, researchers have aimed to use a self‐assembly and self‐delivery methods to construct a phytosterols delivery systems. Next, we will summarize their mechanisms and features related to phytosterols.

#### 3.6.1 Structural Commonality Related to Self‐Assembly Mechanism

**Figure 8:**
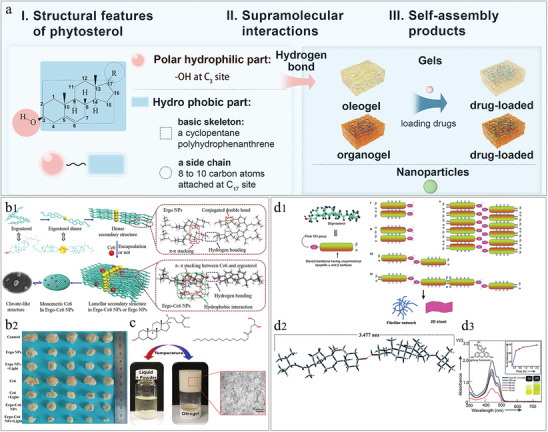
a) Schematic representation of the self‐assembly mechanism of phytosterols: from structural features to supramolecular interactions to products. b_1_) Stacking models of the self‐assembly behavior, show that ergosterol first forms dimers through hydrogen bonding, and then through *π*–*π* stacking to form ergosterol nanoparticles (Ergo NPs), which are loaded with chloretin e6 (Ergo‐Ce6 NPs) through *π*–*π* stacking and hydrophobic interactions. Reproduced with permission.^[^ ^168^ ^]^ Copyright 2019, ACS. b_2_) Photographs of tumor tissue excised after 14 days of treatment in mice. Reproduced with permission.^[^ ^168^ ^]^ Copyright 2019, ACS. c) Microstructure and Images of β‐sitosterol (top left) and glyceryl monostearate (top right) co‐assembled oleogels. Reproduced with permission.^[^ ^173^ ^]^ Copyright 2017, Wiley. d_1_) Schematic representation of the self‐assembly mode of stigmasterol. Hydroxyl groups take part in hydrogen bonding and hydrophobic surfaces take part in van der Waals interactions. Reproduced with permission.^[^ ^179^ ^]^ Copyright 2020, RSC. d_2_) Two stigmasterol molecules form a dimeric structure through hydrogen bonding with a length of 3.47 nm. Reproduced with permission.^[^ ^179^ ^]^ Copyright 2020, RSC. d_3_) Plot of the percentage of carboxy‐fluorescein released from stigmasterol supramolecular gels versus the corresponding experimental time. Reproduced with permission.^[^ ^179^ ^]^ Copyright 2020, RSC.

The following structural properties are considered to be our focus for understanding the self‐assembly mechanisms and strategies of phytosterols in NPHM: I) Polar hydrophilic part: hydroxyl group at C_3_ site. II) Nonpolar lipophilic part: The basic skeleton of cyclopentane polyhydrophenanthrene and a side chain consisting of 8 to 10 carbon atoms attached at position C_17_.^[^ ^166^ ^]^ III) Planar configuration: A large lipophilic steroidal planar structure.^[^ ^167^ ^]^ For the self‐assembly of phytosterols, the hydroxyl groups involved in the formation of hydrogen bonds play a pivotal role in driving the self‐assembly.^[^ ^167^ ^]^ Based on the structural features and supramolecular driving force of phytosterols, phytosterol self‐assembly mainly forms supramolecular materials represented by nanoparticles and organogels, as summarized next (**Figure** **8**a).

#### 3.6.2 Phytosterols Self‐Assembly Products: Nanoparticles

Various phytosterols have been reported to have the ability to self‐assemble to form nanoparticles, such as ergosterol, β‐sitosterol, and stigmasterol. Furthermore, the ability of self‐assembled phytosterol nanoparticles to be used as active delivery carriers for the delivery of other drugs is noteworthy. For example, ergosterol self‐assembles into nanoparticles by first forming dimers through hydrogen bonding and then periodically aggregating vertically and planarly through *π*–*π* interactions (Figure 8b_1_). Experimental results showed that ergosterol self‐assembled nanoparticles not only exerted anticancer effects, but also acted as drug delivery carriers to encapsulate the photosensitizer chloretin e6, which provided synergistic antitumor effects (Figure 8b_2_).^[^ ^168^ ^]^

#### 3.6.3 Phytosterols Self‐Assembly Products: Organogels

##### Oleogels

Oleogels are oil‐structured systems made from gelators added to edible oils so that liquid vegetable oils can be converted into gels.^[^ ^169^ ^]^ It is an ideal alternative to solid animal fats that are rich in unsaturated fatty acids and trans fatty acids that pose a range of hazards to the human cardiovascular system.^[^ ^170^ ^]^ Therefore, oleogels will play an important role in human food health.^[^ ^171^ ^]^ Currently, phytosterols, with their natural source of safety and superior bioactivity, have become one of the most favored gelators by researchers among the low molecular weight gelators in the oleogel system.^[^ ^169^, ^172^ ^]^ Hydrogen bonding is a key driver for phytosterols to promote the self‐assembly of oleogels, e.g., stigmasterol promotes rapeseed oil to form an oleogel by forming a self‐assembled 3D network through the intermolecular hydrogen bonds between hydroxyl groups.^[^ ^167^ ^]^ However, for β‐sitosterol, although it also forms oleogels, the crystals tend to aggregate and precipitate and are not stable enough. Thus, researchers have tried multicomponent co‐assembly to solve the problem.^[^ ^173^ ^]^ For example, β‐sitosterol and γ‐sitosterol can form cylindrical tubules through intermolecular hydrogen bonding, interconnecting to form a self‐assembled fibrous network structure,^[^ ^174^ ^]^ which exhibits excellent oil‐binding capacity, complex modulus, and apparent viscosity.^[^ ^175^ ^]^ Bot et al. also reported the self‐assembly of various phytosterols such as stigmasterol, sitosterol, ergosterol, and cholesterol with γ‐oryzanol to form Sterol + γ‐Oryzanol‐Based oleogels.^[^ ^176^ ^]^ Yang et al. attempted the formation of an oleogel by the co‐assembly of monoglycerides and β‐sitosterol, and showed that more hydrogen bonds were formed and the system had a better ability to regulate the release of volatiles compared to single‐component assembly (Figure 8c).^[^ ^173^ ^]^ Additionally, oleogels have the ability to dissolve, stabilize, and deliver hydrophobic drugs (Figure 8a). For example, Li et al. reported that β‐sitosterol and lecithin were co‐assembled into a supramolecular network to form an oleogel, which was used as a delivery vehicle for the insoluble molecule curcumin, improving the oxidation stability and bioaccessibility of curcumin.^[^ ^177^ ^]^

##### Other Organogels

The structure of phytosterols is hydrophobic, so there are still no findings that any phytosterols can self‐assemble into hydrogels. It's interesting to note that phytosterols can function as gelators in organic systems, encouraging the creation of self‐assembling organogels. For instance, the first sterol natural product gelators to be identified were diosgenin and ergosterol, which broadened the category of low molecular weight gelators.^[^ ^178^ ^]^ Later, in an investigation into the delivery of self‐assembled organogels of phytosterols, Braja Gopal Bag et al. discovered that stigmasterol was capable of self‐assembling in 13 organic solvents to create thermally reversible, mechanically robust gels, a process that begins with the formation of a dimer by hydrogen bonding between the hydroxyl groups of two stigmasterol (Figure 8d_2_), followed by the formation of a gel in the presence of hydrogen bonding and van der Waals forces (Figure 8d_1_). In addition, the gel can be used to load anticancer drugs such as fluorescent agents and DOX (Figure 8d_3_).^[^ ^179^ ^]^

To summarize the significance of phytosterols from their structure to supramolecular materials, the following key points deserve attention. First, the structure and supramolecular interactions highlight the crucial role of the hydrophilic hydroxyl group in promoting hydrogen bonding, which facilitates the formation of nanoparticles, gels, and organogels that share a common key structural component. Second, owing to their pronounced hydrophobicity, phytosterols primarily manifest as nanoparticles and organogels. Notably, phytosterols are the main category in NPHM that form supramolecular oleogels. Lastly, in the realm of applications, research on phytosterol‐based supramolecular bioactive materials is still nascent, primarily focusing on drug delivery and tumor therapy. However, given the wealth of bioactive properties inherent in phytosterols, their potential applications in the medical field remain vast and await further exploration and enhancement.

### 3.7 Self‐assembly of Terpenoids

Terpenoids, a family of NPHMs with isoprene (C5 unit) as the fundamental structural unit, are widely distributed in plants.^[^ ^180^ ^]^ Based on the amount of isoprene, terpenoids can be further divided into terpenoids, diterpenes, and monoterpenes.^[^ ^181^ ^]^ Terpenoids have a wide range of biological activities such as anticancer, treatment of osteoporosis treatment of osteoporosis,^[^ ^182^ ^]^ anti‐inflammatory,^[^ ^183^ ^]^ antibacterial,^[^ ^184^ ^]^ effects on the nervous system,^[^ ^185^ ^]^ and others.

#### 3.7.1 Structural Commonality Related to Self‐Assembly Mechanism

**Figure 9:**
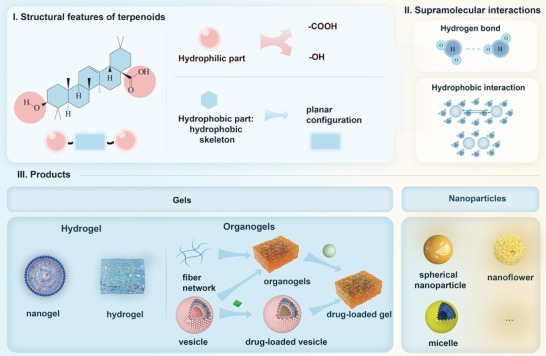
Schematic representation of terpene self‐assembly strategies: from structural characterization, typical supramolecular forces, to supramolecular nanoparticles, hydrogels, and organogels.

**Figure 10:**
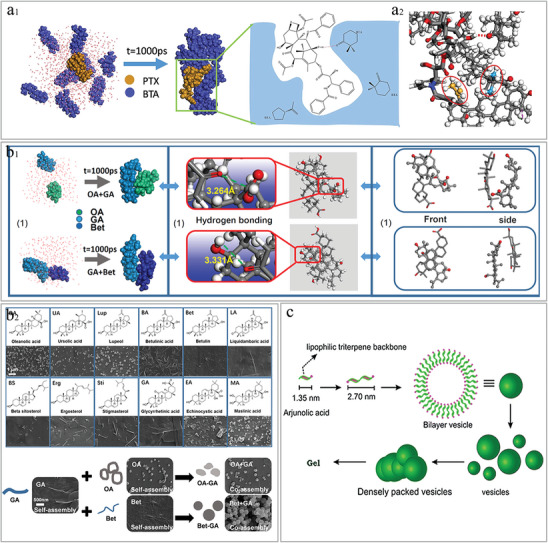
a_1_) Pattern diagram of BTA (Betulonic acid) and PTX (paclitaxel) assembled through hydrogen bonding interactions. Reproduced with permission.^[^ ^189^ ^]^ Copyright 2020, Elsevier. a_2_) Molecular simulations of TA and PTX stereoconformation with hydrophobic interactions present during assembly (red circles). Reproduced with permission.^[^ ^189^ ^]^ Copyright 2020, Elsevier. b_1_) Analysis of the co‐assembly mechanism of olanolic acid (OA)/glycyrrhetinic acid and betulin (Bet)/ glycyrrhetinic acid. The red squares represent hydrogen bonds and the blue squares represent hydrophobic levels. Reproduced with permission.^[^ ^188^ ^]^ Copyright 2020, ACS. b_2_) Above: detailed structure and SEM image of the supramolecular product of the self‐assembled terpenoids of NPHM. Bottom: SEM image of OA/ GA and Bet/GA that can co‐assemble to form homogeneous nanospheres. Reproduced with permission.^[^ ^188^ ^]^ Copyright 2020, ACS. c) Schematic representation of the terpenoid arjunolic acid (length 1.35 nm) first self‐assembling into vesicles (membrane thickness 2.7 nm) and then forming a gel. Reproduced with permission.^[^ ^207^ ^]^ Copyright 2014, RSC.

The following structural properties are suggested to be our focus for understanding the self‐assembly mechanisms and strategies involved in the terpenoids of NPHMs: I) There are many chiral centers present.^[^ ^186^ ^]^ II) Hydrophilic part: hydroxyl and carboxyl groups in different numbers and positions and with different orientations.^[^ ^186^ ^]^ III) Hydrophobic part: hydrophobic skeleton.^[^ ^187^ ^]^ IV) Planar molecular conformation. In conclusion, the amphiphilic structure and planar conformation of terpenoids promote hydrophobic interactions,^[^ ^188^ ^]^ and the hydrophilic portion facilitates hydrogen bonding, thus hydrogen bonding and hydrophobic interactions are the main drivers of their self‐assembly (**Figure** **9**).^[^ ^189^ ^]^ For instance, the co‐assembly of two terpenoids NPHMs, betulonic acid, and paclitaxel, is primarily driven by hydrogen bonding and hydrophobic interactions (**Figure** **10**a).^[^ ^189^ ^]^ Similarly, among the terpenoid NPHMs including oleanolic acid, glycyrrhetinic acid, and betulin, when oleanolic acid co‐assembles with glycyrrhetinic acid, or betulin with glycyrrhetinic acid, the spatial orientation of their molecular backbones assumes a planar configuration (front view) and parallel planes (side view). This alignment facilitates the formation of hydrophobic interactions between the molecular backbones, ultimately leading to a more stable structure (Figure 10b_1_).^[^ ^188^ ^]^ Additionally, the presence of hydrophilic components further contributes to the formation of hydrogen bonds, further promoting co‐assembled structure.^[^ ^188^ ^]^In addition, for terpenoids with benzene rings, *π*–*π* interactions also plays a role.^[^ ^190^ ^]^ Terpenoids can self‐assemble into a variety of products, primarily nanoparticles, micelles, nanofibers,^[^ ^191^ ^]^ nanogels,^[^ ^192^ ^]^ hydrogels,^[^ ^193^ ^]^ and organogels, as a result of the aforementioned structural characteristics. Subsequently, we will accentuate and succinctly outline their distinctive attributes.

#### 3.7.2 Terpenoids Self‐Assembly Products: Nanoparticles and Gels

The supramolecular materials of terpenoid primarily consist of three types: hydrogels, organogels, and nanoparticles (Figure 9). Terpenoid self‐assembled nanoparticles exhibit several notable features worthy of our attention. First, in terms of composition, a diverse range of terpene NPHM can form nanoparticles with various morphologies—such as hollow spherical, solid spherical,^[^ ^188^ ^]^ rod‐like,^[^ ^190^ ^]^ flower‐like^[^ ^194^ ^]^ shapes—through direct self‐assembly or co‐assembly with other terpene NPHM (Figure 10b_2_). Second, functionally speaking, specific terpene supramolecular nanoparticles have demonstrated an ability to regulate cellular functions via direct interactions between nanomaterials and organisms. For instance, Luo and colleagues discovered that oleanolic acid self‐assembled micelles can directly bind to the 20S proteasome subunit alpha 6, dynamically modulating the conformational changes of its N‐terminal domain and controlling the influx of proteins into the 20S proteasome, ultimately promoting pyrolysis in cancerous cells.^[^ ^195^ ^]^ Furthermore, these terpenoid‐based supramolecular nanoparticles serve as effective delivery vehicles, enhancing the solubility of other drug,^[^ ^196^ ^]^ reducing their toxicity.^[^ ^188^ ^]^ Lastly, nanoparticles co‐assembled from terpenoids and other molecules—such as the photosensitizer chlorin e6,^[^ ^197^ ^]^ curcumin,^[^ ^198^ ^]^—exhibit remarkable synergistic effects. As an example, Zhao and his team successfully co‐assembled the anticancer terpenoid ursolic acid with the hepatocyte‐targeting molecule lactobionic acid and the photosensitizer indocyanine green into nanoparticles. These nanoparticles not only possess tumor‐targeting properties but also exhibit fluorescence imaging capabilities and significant antitumor efficacy.^[^ ^199^ ^]^ Additionally, the co‐assembly of the terpenoid luteolin and the angiogenesis inhibitor nintedanib into nanoparticles demonstrates multifaceted effects, including anti‐angiogenesis, anti‐fibrosis, anti‐inflammatory, and anti‐oxidative properties, in the treatment of choroidal neovascularization.^[^ ^14^ ^]^

Due to the hydrophobicity of terpenoids, only a limited number of terpenoids have the capacity to spontaneously assemble and form hydrogels, as hydrogel formation necessitates a delicate balance between hydrophilicity and hydrophobicity. An example of such a self‐assembled hydrogel is the glycyrrhetinic acid hydrogel developed by Zou et al. The previous section on saponins focused on summarizing GA‐based hydrogel systems, and the GA structure with two glucuronides removed is glycyrrhetinic acid. The glycyrrhetinic acid hydrogel is formed through the interaction of *π*–*π*, hydrogen bonding, and dipole‐dipole forces. It can be sprayed and functions as a physical barrier material to reduce the occurrence of peritoneal adhesions after surgery. This is achieved by reducing inflammatory reactions and the deposition of collagen fibers.^[^ ^193^ ^]^ In addition, nanogels are another form of terpenoid gels. The self‐assembled oleanolic acid nanogel reported by Li et al. is characterized by elasticity tunable as well as stronger anti‐tumor effects than nanoparticles (Figure 9).^[^ ^192^ ^]^

Another type of terpenoids supramolecular gel is the organogel, which exhibits a unique gelation mechanism: some form gels by self‐assembling fibers,^[^ ^200^ ^]^ and others can self‐assemble into bilayer vesicles to form gels (Figure 9).^[^ ^201^ ^]^ I) Organogels formed through 3D fiber networks: In a range of organic solutions or alcohol and water mixes, terpenoids can self‐assemble to produce an organogel, which is made up of a network of fibers with a composition of nano‐ to micrometer‐length fibers.^[^ ^200^, ^202^ ^]^ Two primary characteristics of this organogel are worth mentioning: One advantage is its capacity to create gels when mixed with ethanol and water, which indicates superior biocompatibility compared to many materials that can only form gels in harmful chemical solvents.^[^ ^203^ ^]^ Furthermore, terpenoids organogels have demonstrated remarkable efficacy in delivering pharmaceuticals as bioactive gel scaffolds (Figure 9). For instance, Zhi et al. discovered that liquidambaric acid formed self‐assembled gels in a solution containing a mixture of ethanol and water, which can be used as ab injectable gel scaffolds to load DOX and were found to have a combined anticancer effect by regulating cyclooxygenase‐2 expression and decreasing prostaglandin E2 conten.^[^ ^203^ ^]^ II) Organogels formed through vesicles: the ingenuity of this structure lies in the fact that the drug can be encapsulated within the terpenoid‐formed vesicles and then delivered through the gel (Figure 9). For example, rhodamine B, the anticancer drug DOX, and many other fluorophores can be encapsulated in self‐assembled bilayer vesicle self‐assemblies formed by arjunolic acid, ursolic acid, oleanolic acid, and so on, and then the supramolecular gel is formed by the bilayer vesicles.^[^ ^204^ ^]^ Ursolic acid self‐assembled gels possess the capacity to specifically release anticancer medicines in the acidic conditions of the tumor microenvironment.^[^ ^205^ ^]^ Additionally, they demonstrate favorable iodine adsorption characteristics owing to their extremely porous structure.^[^ ^206^ ^]^ The Arjunolic acid self‐assembling gel exhibits drug release under normal physiological pH conditions (Figure 10c).^[^ ^207^ ^]^

In summary, the hydrophobic and hydrophilic structures of terpenoids allow for self‐assembly, leading to the formation of products such as nanoparticles and gels, especially hydrogels and vesicular gels. At the same time, the biological activity and multiple forms of terpenoids as carriers can be used to control drug release and delivery in a variety of ways, and thus have great potential as carriers for specialized drug delivery.

## 4 Supramolecular Interactions and Preparation Methods of NPHM Self‐Assembly

The previous section summarized in detail the representative structural features of each of the seven major classes of NPHM, while highlighting which supramolecular driving force species the NPHM self‐assembly process tends to rely on under different structural features. In this section, a concise and integrated overview of all the supramolecular forces that primarily drive NPHM self‐assembly will first be presented, with a brief indication of the important roles that each driver plays in NPHM. This will be followed by an overview of the main preparation methods according to the different types of supramolecular materials. Self‐assembly of NPHM refers to the process where NPHM, as structural motifs, spontaneously transition from a disordered state to an ordered state through non‐covalent interactions.^[^ ^208^ ^]^ This process does not form covalent bonds to connect the structural motifs together, but is able to move and regulate the positions,^[^ ^209^ ^]^ thus providing mobility, tunability and reversibility between the structural motifs.^[^ ^210^ ^]^ In summary, NPHM monomers are neither simply added to each other nor tightly connected through chemical bonds, but are attracted to each other through multiple non‐covalent interaction forces to form an ordered NPHM‐monomer‐based supramolecular product that exhibits properties and functions that are not available in NPHM monomers.

### 4.1 Supramolecular Interactions

**Figure 11:**
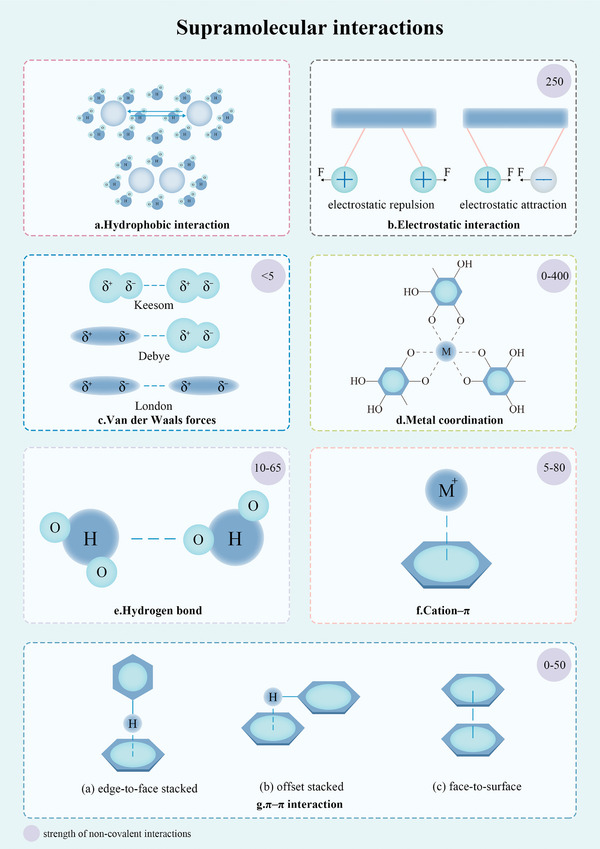
Types of major supramolecular interactions that drive the NPHM self‐assembly process, including hydrophobic interactions, electrostatic interactions, van der Waals forces, metal‐ligand interactions, hydrogen bonding, cation‐π, and *π*–*π* interactions. The purple circle in the upper right corner indicates the strength of this supramolecular interaction. Reproduced with permission.^[^ ^218^ ^]^ Copyright 2020, ACS.

The key driving force of self‐assembly is supramolecular non‐covalent interactions. Covalent bonds are stable, irreversible, and dictate the arrangement of atoms in a molecule (primary structure).^[^ ^211^ ^]^ In contrast, non‐covalent bonds, first, are weaker than covalent interactions ≈10–100 times, including van der Waals forces of less than 5 kJ mol^−1^, hydrogen bonds of ≈10–65 kJ mol^−1^, etc.^[^ ^212^ ^]^ Second, non‐covalent bonds are reversible, which implies a certain stimulus responsiveness. Third, non‐covalent bonds mainly affect the tertiary and quaternary structure of molecules, the conformation, aggregation, and specific properties.^[^ ^211^ ^]^ The five most common noncovalent interactions during NPHM self‐assembly include the following. I) Hydrophobic interaction: It happens when nonpolar elements avoid contact with a polar environment like water by forming aggregates. Hydrophobic interactions are important drivers for the self‐assembly of amphiphilic NPHM saponins to form supramolecular products such as micelles and vesicles. For saponins in NPHM with an amphiphilic structure that is hydrophobic at one end and hydrophilic at the other, this typical structure is able to self‐assemble through hydrophobic interactions to form representative products of micelles and vesicles. II) Electrostatic interaction: Electrostatic effects include electrostatic attraction and electrostatic repulsion. This results in identically charged molecules and groups repelling each other, while oppositely charged molecules and groups attract each other closer together. This is critical for the self‐assembly of NPHM with positively charged groups, such as berberine, and negatively charged NPHM by attracting them through electrostatic interactions. In addition, the phenomenon that pH affects the formation of some NPHM self‐assembly products is also related to the electrostatic effect, because pH can determine the protonation and deprotonation of some groups (e.g., carboxyl and hydroxyl) of NPHM, which in turn affects the self‐assembly through the electrostatic effect between the protonated and unprotonated groups.^[^ ^213^ ^]^ III) Metal–ligand coordination: Metal coordination exist between the metal ion as the coordination center and the surrounding array of organic molecules as the ligand.^[^ ^214^ ^]^ For example, polyphenols is exemplary representative of NPHM capable of forming metal‐NPHM coordination networks through metal coordination. IV) Hydrogen bonding: Hydrogen bonding refers to the interaction between a hydrogen atom joining two electronegative atoms, usually found in the A‐H…B system, such as NH…O, NH…N, OH…N, CH…O hydrogen bonds.^[^ ^215^ ^]^ Hydrogen bonds play a crucial role in in the self‐assembly of NPHM and are intimately involved in dimer formation.^[^ ^216^ ^]^ V) *π*–*π* interactions: *π*–*π* interactions refers to non‐covalent interactions between aromatic rings containing π‐orbital, which are categorized as edge‐to‐face stacked, offset stacked, and face‐to‐surface,^[^ ^217^ ^]^ and are widely found in the self‐assembly of NPHM containing π‐systems (**Figure** **11**).

In summary, during the self‐assembly of NPHM, the above supramolecular interactions are often not driven singly, but rather multiple together, thus facilitating the formation of nanostructures with complex, advanced dimensions.

### 4.2 Preparation Methods

**Figure 12:**
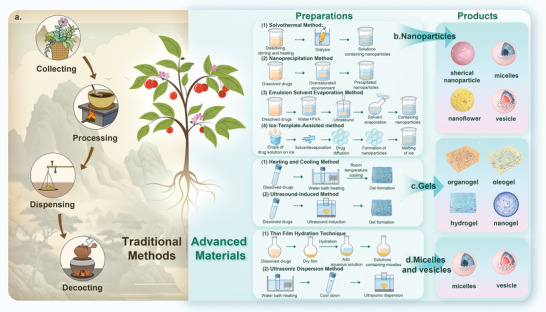
Evolution of human utilization of herbs: from traditional methods to advanced techniques and materials. a) Traditional steps in the utilization of herbs medicines for the treatment of diseases. b) Preparations of NPHM‐based supramolecular nanoparticles. c) Preparations of NPHM‐based supramolecular gels. d) Preparation of NPHM‐based supramolecular gels.

Over the past thousands of years, human beings have often had to follow a series of complex steps including collecting, processing, dispensing and decocting in the utilization of herbal medicines (**Figure** **12**a). With the booming development of modern science and technology, the emergence of new technologies and new preparation means has revolutionized the utilization of herbal medicines. This section will mainly summarize the advanced preparation means of NPHM‐based supramolecular advanced materials, which have opened a whole new chapter in the application of herbal medicine.

#### 4.2.1 NPHM‐Based Supramolecular Nanoparticles

NPHM can self‐assemble to form nanoparticles of various shapes, including nanospheres, nanoflowers, and nanotubes. Examples of several preparation methods for NPHM self‐assembled nanoparticles: I) solvothermal method: first, the drug is dissolved, then heated and stirred, followed by dialysis of the solution with deionized water or phosphate buffer saline (PBS) to obtain a solution containing self‐assembled nanoparticles.^[^ ^92^, ^95^ ^]^ II) emulsion solvent evaporation method: The drug was first dissolved in an organic solvent, then the organic phase was added to an aqueous PVA solution and sonicated with an ultrasonic probe. Then the emulsion was added to the aqueous PVA solution, and the product was obtained by stirring at room temperature, centrifugation, washing and lyophilization.^[^ ^219^ ^]^ III) Nanoprecipitation method: The drug is first dissolved in an organic solvent and then added to water under vigorous stirring to form a supersaturated environment, resulting in nanoparticle precipitation. Finally, the organic solvent is removed by dialysis, bubbling, ultrafiltration, freeze‐drying.^[^ ^220^ ^]^ IV) The drug is first dissolved in an organic solvent and then dripped onto the surface of an ice template. After the organic solvent evaporates, the drug‐loaded ice template is kept at −20 °C for 24 h. Subsequently, the drug‐loaded ice template is either thawed or freeze‐dried (Figure 12b).^[^ ^221^ ^]^

In addition, NPHM with hydrophobic at one end and hydrophilic at the other structural features can also form micelles or vesicles, a class of nanoparticles with hollow structural structural features that can be loaded with various hydrophobic drugs. Commonly used methods for the preparation of natural small molecule supramolecular micelles and vesicles are I) thin film hydration technique: thin film hydration technique is frequently employed in the preparation of self‐assembled micelles and vesicles using NPHMs. This method entails dissolving amphiphilic NPHM and the desired drug in an organic solvent within a round‐bottomed flask of a rotary evaporator. Following the evaporation of the organic solvent, a dry film is formed at the flask's bottom. Subsequently, water is introduced to the film at a temperature surpassing the surfactant transition temperature and agitated, followed by sonication to achieve micelles or vesicles of uniform size.^[^ ^222^ ^]^ II) ultrasonic dispersion method: the amphiphiles were dissolved in preheated distilled water and then the drug to be encapsulated was added. The mixture was incubated in a water bath at 60 °C for 1 h to form self‐assembled micelles, which were subsequently cooled to room temperature and then the resulting preparation was further dispersed using an ultrasonic homogenizer to finalize the micellar solution (Figure 12d).^[^ ^127^ ^]^

#### 4.2.2 NPHM‐Based Supramolecular Gels

NPHM are unique small‐molecule gelling agents that have the ability to generate many types of supramolecular gels by self‐assembly. These include nanogels, hydrogels, oleogels, and organogels. Among them, NPHM‐based supramolecular hydrogel has the widest biological applications due to its high biocompatibility, injectability, and self‐repair ability. Commonly used methods for the preparation of natural small molecule supramolecular hydrogels are I) heating and cooling method:in short, the drug powder is added to a solution and heated in a water bath, then left to cool at room temperature to form a gel.^[^ ^223^ ^]^ II) ultrasound‐induced method: This refers to the promotion of dissolution, homogenization, and gel formation induced by ultrasound. For example, in the preparation of rhein hydrogels by Zheng et al, rhein was dissolved in PBS and then induced by ultrasound to obtain a homogeneous and stable hydrogel at room temperature (Figure 12c).^[^ ^11^ ^]^

## 5 Biomedical Applications

In this section, the prospects of biomedical applications of NPHM‐based supramolecular biomaterials will be discussed in six aspects: disease treatment, toxicity reduction, synergism, drug delivery vehicles with therapeutic effects, optimized dosage forms, and penetration capabilities.

### 5.1 Disease Treatment: Powerful Pharmacological Activity Inherited from NPHM

**Figure 13:**
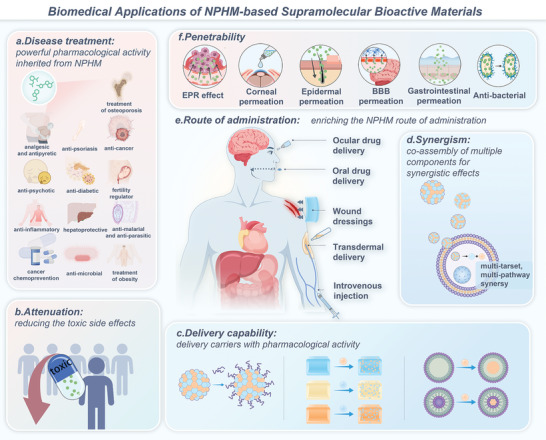
Schematic representation of biomedical applications of NPHM‐based bioactive supramolecular materials including disease treatment, toxicity reduction, synergism, drug delivery vehicles with therapeutic effects, optimized dosage forms, and penetration capabilities. Created with BioRender.com.

NPHM‐based supramolecular bioactive materials are unique in that they are composed entirely of pharmacologically active NPHM. Therefore, clarifying the main therapeutic applications of NPHMs will enable us to understand the most important potential of NPHM‐based supramolecular materials. However, in this review, a detailed description of the specific mechanisms of NPHM and NPHM‐based supramolecular bioactive materials will not be pursued, as a large number of reviews have already summarized the therapeutic mechanisms of NPHM.^[^ ^224^ ^]^ Although NPHMs were categorized into seven classes based on self‐assembly strategies in the previous section, it is not advisable to strictly separate the therapeutic scope of these classes from a disease perspective. This is because their therapeutic applications often intersect and are not mutually exclusive. Therefore, classifying NPHM according to therapeutic purpose is a more appropriate choice. According to Lutfun Nahar et al. NPHMs excel in the following 13 classes of diseases:1) analgesic and antipyretic, 2) anticancer, 3) antidiabetic, 4) anti‐inflammatory, 5) antimalarial and antiparasitic, 6) antimicrobial, 7) antipsoriasis, 8) antipsychotic, 9) fertility regulator, 10) hepatoprotective, nephroprotective and neuroprotective effects, 11) cancer chemoprevention, 12) treatment of obesity, and 13) treatment and prevention of osteoporosis.^[^ ^225^ ^]^ Currently, NPHM‐based supramolecular bioactive materials have been reported mainly in the fields of brain disorders,^[^ ^226^ ^]^ eye conditions,^[^ ^126b^ ^]^ wound healing,^[^ ^57^ ^]^ cardiac and renal protection,^[^ ^17^, ^158^ ^]^ intestinal disorders,^[^ ^70^ ^]^ antitumor,^[^ ^227^ ^]^ arthritis,^[^ ^61^ ^]^ and antibacterial applications,^[^ ^228^ ^]^ with many potential applications and roles to be further explored and enriched (**Figure** **13**a).

In summary, when selecting appropriate NPHM for disease treatment, researchers are suggested not to limit themselves to the range of diseases currently addressed by NPHM‐based supramolecular materials. Instead, they can select suitable NPHM based on the needs of various diseases and explore different self‐assembly strategies.

### 5.2 Attenuation: Reducing the Toxic Side Effects

Non‐covalent interactions drive the self‐assembly process, potentially allowing toxic groups in a molecule to rearrange and interact differently, thereby reducing toxicity by forming supramolecules. For example, aristolochic acid is a NPHM that can lead to liver cancer and acute kidney injury. However, when it co‐assembled with berberine into a linear heterogeneous supramolecule, it shields the harmful components of aristolochic acid, such as nitro and hydroxyl groups, from converting into toxic metabolites like aristolactam and DNA adducts. This process effectively reduces the nephrotoxic effects of aristolochic acid.^[^ ^17^ ^]^ In addition to mitigating the toxicity of those toxic NPHM, many NPHM‐based supramolecular bioactive materials can also play a palliative role against other drug‐induced toxicities, such as protective and mitigating effects against DOX‐induced apoptosis,^[^ ^229^ ^]^ cardiotoxicity,^[^ ^123^, ^158^ ^]^ and other side effects (Figure 13b).

### 5.3 Synergism: Co‐Assembly of Multiple Components for Multi‐Pathway Therapy

NPHM‐based supramolecular bioactive materials can be formed by self‐assembly of multiple NPHM with different biological properties through non‐covalent interactions. Since the non‐covalent interactions are weak and do not change the molecular structure of each NPHM, the components can be released individually when subjected to a specific stimulus to exert a therapeutic effect. This property facilitates the binding of pharmacological components with different mechanisms, which can occur between NPHM and NPHM, as well as between NPHM and other functional monomers, and the supramolecular product enhances the original therapeutic efficacy of the monomers as well as the synergistic efficacy of each monomer. For example, the self‐assembly of oleanolic acid with chlorin e6 to form nanoparticles resulted in a 23‐fold increase in the accumulation of chlorin e6 in cancer cells, exerting synergistic chemotherapeutic and acousto‐optic anti‐tumor effects.^[^ ^197^ ^]^ Co‐assembly of ursolic acid, hepatocyte‐targeting molecule, and photosensitizer indocyanine green to form nanoparticles for combined cancer imaging and chemo‐photo therapy.^[^ ^199^ ^]^ Norcantharidin, Cu^2+^ and GA can be co‐assembled into a three‐component self‐assembled hydrogel. Norcantharidin triggers apoptosis, Cu^2+^ catalyzes the fenton reaction to produce reactive oxygen species, and GA acts as an anti‐inflammatory agent. Together, these components work synergistically to target multiple pathways and provide an anti‐tumor effect (Figure 13d).^[^ ^16^ ^]^

In summary, depending on the needs of the disease, researchers can select different NPHMs or functional molecules for co‐assembly attempts, therefore achieving synergistic treatment of the disease.

### 5.4 Delivery Capability: Delivery Carriers with Pharmacological Activity

Synthesis and design of ideal drug delivery carriers are the focus of attention in the field of drug delivery. However, how to achieve ideal delivery and therapeutic effects while avoiding side effects of the carriers is a key pursuit. NPHM‐based supramolecular bioactive materials provide a good idea for this goal. Not limited to the self‐delivery of NPHM, NPHM‐based supramolecular bioactive materials have the potential to be used as drug delivery carriers for a wide range of drugs, including, e.g., glyburide,^[^ ^230^ ^]^ diclofenac,^[^ ^126b^ ^]^ rhodamine B, DOX,^[^ ^204^ ^]^ paclitaxel,^[^ ^129^ ^]^ paeoniflorin,^[^ ^124a^ ^]^ and so on. Although, compared with other carriers, it is still not mature enough in terms of drug loading rate and stability etc., the ability of this delivery vehicle to exert the biological activity of NPHM is its greatest value (Figure 13c).

In conclusion, NPHM‐based supramolecular bioactive materials bring a new class of delivery vehicles with strong pharmacological activity to the drug delivery field, which signifies the great potential of drug delivery vehicles to exert pharmacodynamic effects together with the delivered drugs. We are optimistic that NPHM‐based supramolecular bioactive materials will be an innovative and unique drug carrier that will have an impact on drug delivery in the future.

### 5.5 Improved Dosage Forms: Enriching the NPHM Route of Administration

For the purposes of improving bioavailability, reducing side effects, meeting different clinical needs, and improving patient compliance, the optimization of NPHM dosage forms is necessary. A series of NPHM‐based supramolecular bioactive materials have been formed to largely enrich the routes of administration of NPHM, including intravenous injection, oral delivery, transdermal delivery, localized delivery in the cornea, wounds, brain and other parts of the body. For example, NPHM‐based supramolecular nanoparticles, injectable gel scaffolds, and other forms of NPHM promote the pharmacological efficacy of NPHM for intravenous injection such as slow release and permeation. Second, environmentally sensitive NPHM‐based biomaterials help to realize a localized drug release system that aids in breaking through the limitations of oral drug delivery by gastrointestinal pH. For example, Jia et al. reported an NPHM baicalin self‐assembled with Al^3+^ into nanoparticles, which is well able to play different responsiveness to gastric and small intestinal environments for oral drug delivery. Specifically, it was able to produce stronger binding under gastric acidic conditions (pH = 1.2) and very weak binding under intestinal alkaline conditions (pH = 7.8), thus this acid‐base responsive behavior helps to prevent premature drug release in the stomach and achieve release in the intestine.^[^ ^231^ ^]^ Additionally, some NPHM‐based supramolecular nanoparticles can enable intravenous formulations to transition to transdermal drug delivery by penetrating the skin barrier. Finally, to address side effects such as systemic toxicity from oral or intravenous administration, NPHM‐based supramolecular bioactive materials are crucial for local drug delivery. Through self‐assembly, it forms supramolecular products like hydrogels, which have already been applied in scenarios such as in situ injections in bones and joints, in situ gelation for cutaneous wounds,^[^ ^232^ ^]^ local drug delivery for traumatic brain injury,^[^ ^226^ ^]^ and in situ delivery to the cornea. Local delivery of NPHM not only increases the drug concentration at the target site but also reduces toxic side effects, ensures rapid onset of action, and avoids first‐pass metabolism (Figure 13e).

Overall, NPHM‐based supramolecular bioactive materials offer various drug delivery methods to meet clinical needs. Additionally, some delivery modes, such as inhalation, mucosal, and nasal routes, have yet to be explored in this field, presenting opportunities for further research.

### 5.6 Permeation: Permeability of NPHM‐Based Bioactive Materials

Biological barriers, including the blood‐brain barrier (BBB), corneal barrier, epidermal barrier, and intestinal barrier, serve a crucial protective function while concurrently posing a challenge in enabling drugs to reach the disease site for therapeutic efficacy. As a pivotal strategy for drug delivery, the development of materials that can penetrate biological barriers and efficiently deliver drugs to the targeted disease site holds significant promise for therapeutic effects. Certain NPHM‐based nanoparticles have demonstrated the remarkable ability to penetrate biological barriers, even without requiring the artificial modification of ligands on their surfaces. Concurrently, numerous studies have highlighted the therapeutic benefits of NHMP in addressing a wide range of related conditions, encompassing brain diseases^[^ ^233^ ^]^ (e.g., Alzheimer's disease,^[^ ^234^ ^]^ Multiple Sclerosis,^[^ ^235^ ^]^ Parkinson's disease,^[^ ^236^ ^]^ Stroke,^[^ ^237^ ^]^ Gliom^[^ ^238^ ^]^), intestinal diseases^[^ ^239^ ^]^ (e.g., acute colitis,^[^ ^240^ ^]^ ulcerative colitis,^[^ ^162^ ^]^ diarrhea‐predominant irritable bowel syndrome^[^ ^157a^ ^]^), ophthalmic diseases^[^ ^241^ ^]^ (e.g., glaucoma^[^ ^242^ ^]^),and others. While the exploration of NPHM self‐assembling into supramolecular bioactivities for barrier penetration and disease therapy is still in its nascent stages, this section underscores the immense potential of such materials to emerge as dual‐functional entities, capable of both barrier permeation and targeted disease treatment (Figure 13f).

#### 5.6.1 Corneal Barrier Permeation

Because of the static and dynamic ocular barriers that shield the eyes,^[^ ^243^ ^]^ topical drops have an extremely poor bioavailability—less than 5% of the medication passes past the ocular barrier and into the aqueous humor.^[^ ^244^ ^]^ Therefore, improving drug retention and duration of action, as well as increasing the penetration of drugs into the cornea, are two important factors in ocular drug delivery.^[^ ^245^ ^]^ NPHM capable of self‐assembling into ultra‐small supramolecular micelles play a crucial role in corneal permeation. For instance, ginsenoside Rb1, an NPHM with multiple biological activities such as anti‐inflammatory and antioxidant properties, can self‐assemble into ultra‐small micelles (<8 nm). When diclofenac is encapsulated within these micelles, ocular levels increase by 255.43% compared to the diclofenac‐only eye drop group.^[^ ^126b^ ^]^ Similar results were observed in another study. The researchers utilized ultra‐small micelles (<4 nm) formed by the self‐assembly of Rebaudioside A, a hypoglycemic and antihyperlipidemic NPHM, to encapsulate the antioxidant NPHM Astragalus purpureus. These Rebaudioside A micelles increased the ocular concentration of Astragalus purpureus by a factor of 4.99.^[^ ^246^ ^]^ Therefore, the potential application of NPHM supramolecular active materials contributing to corneal permeation is noteworthy, and the mechanism needs to be further investigated. Therefore, the potential of NPHM‐based supramolecular materials to enhance corneal permeation for ocular drug delivery is of great interest, though the underlying mechanisms require further elucidation.

#### 5.6.2 Epidermal Barrier Penetration

Transdermal drug delivery has many benefits, such as bypassing the first metabolism of oral drugs in the liver, maintaining constant blood levels, improving patient compliance, being non‐invasive, and allowing self‐administration.^[^ ^247^ ^]^ However, transdermal drug delivery necessitates the medication to traverse the stratum corneum barrier of the skin, which presents certain needs and obstacles for the drug or the vehicle used for delivery. Cellulose, dextran, acrylic polymers, and polycaprolactone are examples of materials that have been used as carriers for transdermal drug delivery.^[^ ^248^ ^]^ In contrast to materials that are not biologically active, bioactive materials based on saponin‐based NPHM can interact with cell membranes to promote penetration into the stratum corneum. For example, Zou et al. reported a ginsenoside nanoparticle for delivery of insulin. The ginsenoside facilitated the penetration of insulin into the stratum corneum and achieved a sustained release of insulin and maintained a hypoglycaemic efficiency of ≈50% for ≈48 h.^[^ ^123a^ ^]^ However, the research on the application of NPHM supramolecular active materials for transdermal drug delivery is still in its infancy. This area shows great potential, and we eagerly anticipate the development of more nanosystems based on NPHM for transdermal drug delivery in the coming years.

#### 5.6.3 Intestinal Barrier Permeation

The gastrointestinal barrier limits the effectiveness of drug delivery via this route. NPHM‐based supramolecular bio‐nanoparticles possess the dual capabilities of penetrating the gastrointestinal barrier and carrying drugs without modification, making them ideal for oral administration.^[^ ^249^ ^]^ For example, an NPHM dehydrotrimethoprim self‐assembled nanoparticles, derived from medical herbs, can penetrate the gastrointestinal tract via an apical sodium‐dependent bile transporter. Additionally, these nanoparticles can serve as oral carriers for the hypoglycemic drug GLP‐1, which is traditionally administered intravenously.^[^ ^250^ ^]^ Utilizing NPHM‐based supramolecular nanoparticles with gastrointestinal penetration capabilities facilitates the transition of other formulations to oral delivery, enabling dosing with meals and improving patient compliance.

#### 5.6.4 Blood‐Brain Barrier (BBB) Permeation

The BBB is an important protective structure of the nervous system, preventing harmful substances from entering the brain. However, it also limits the entry of drugs into the brain for therapeutic effects. Nanoparticles have size advantages and are able to penetrate the endothelial cells of brain capillaries through actions such asendocytosis and transcytosis,^[^ ^251^ ^]^ thus penetrating the BBB. The NPHM‐based supramolecular nanoparticles have the following benefits: (I) They can penetrate the BBB without the need of artificially modifying the ligand on the surface of the nanoparticles. (II) It has a therapeutic effect on brain diseases without the need to deliver other drugs. (III) Can be used as a delivery vehicle for other drugs for the treatment of central nervous system diseases, facilitating the transport of them across the BBB. For example, betulinic acid extracted from herbal medicine is able to self‐assemble into rod‐like nanoparticles. Without carrying any drug or artificial modification, it can penetrate the BBB via cannabinoid receptor 1‐mediated transcytosis, which then inhibits the production of ROS in hypoxic environments and promotes stroke recovery. Further, it also acts as a delivery vehicle, significantly facilitating the penetration of the stroke drug glyburide into the ischaemic brain.^[^ ^230^ ^]^

#### 5.6.5 Tumor Permeation

The process of neovascularization in tumors is characterized by large fenestrations and reduced lymphatic drainage, which may cause nanoparticles to aggregate inside the tumor via vascular leakage. This is referred to as the enhanced permeability and retention (EPR) effect.^[^ ^252^ ^]^ Under EPR mediation, NPHM‐based supramolecular nanoparticles are passively targeted to tumors and highly enriched to achieve multi‐strategy anti‐tumor therapies including ferroptotic therapy,^[^ ^74^ ^]^ chemotherapy,^[^ ^253^ ^]^ dual‐chemotherapy,^[^ ^110^, ^227^ ^]^ immunotherapy,^[^ ^254^ ^]^ photodynamic therapy,^[^ ^168^, ^255^ ^]^ sono‐photodynamic therapy,^[^ ^197^ ^]^ sonodynamic therapy,^[^ ^256^ ^]^ chemo‐photo combination therapy^[^ ^199^ ^]^ among many other combination therapies.

#### 5.6.6 Microbial Biofilm Permeation

The misuse of antibiotics is leading to a growing and challenging issue of bacterial drug resistance, which has significant implications for combating bacterial infections.^[^ ^228^ ^]^ Hence, the emergence of novel antimicrobial medications is a matter of pressing importance. Natural plants are a valuable source of antibiotics, and many NPHMs possess antibacterial properties. On this basis, the development of supramolecular self‐assemblies of NPHMs can further enhances the antibacterial ability. First, hydrophobic NPHMs can exhibit their hydrophilic portions on the outer layer of the self‐assembled product driven by hydrophobic interactions, while hydrophilic surfaces are more adherent to bacterial surfaces, thus facilitating attachment to bacterial surfaces for sustained release of the drug, e.g., when comparing the antimicrobial capacity of the co‐assembled products of berberine versus those of baicalin or wogonoside, the former showed stronger antimicrobial ability due to the hydrophilic surfaces of the former and the latter hydrophobic surfaces of the former.^[^ ^9^ ^]^ On the other hand, NPHMs has a wide variety of functional groups on the surface of its self‐assembly products that can create affinity and promote attachment and penetration through non‐covalent interactions with bacterial surfaces. For example, rhein not only has an affinity for biofilms with its anthraquinone ring, but also forms hydrogen bonds between its carboxyl, phenolic hydroxyl, and carbonyl groups and the amide and carboxyl groups on the peptidoglycan. This interaction facilitates the attachment of berberine with rhein self‐assembled nanoparticles to S. aureus biofilms, resulting in their cleavage.^[^ ^109^ ^]^ In conclusion, NPHMs self‐assembled biomaterials have strong attachment and penetration removal ability for microbial biofilm, promising as a new class of nano‐antimicrobial agents.

## 6 Conclusion and Perspectives

NPHM, a gift from nature, possesses potent pharmacological activities that can play a therapeutic role in a range of diseases. Currently, NPHM‐based supramolecular bioactive materials, formed through the direct self‐assembly of NPHM, have gradually entered the research field. These materials represent a new and unique class of bioactive materials with renewable components, simple preparation and production processes, high biocompatibility, and strong therapeutic effects. They meet the requirements for environmentally friendly, green chemistry and sustainable human development, and they hold promising potential for the advantageous intersection of medicine and industry in the future, creating significant value.

However, the self‐assembly mechanisms of NPHM vary, and a systematic summary of this system is still lacking. We aim to address this gap by making efforts and contributions to this field. The focus of this review is to categorize NPHMs into the following seven categories based on their structures associated with self‐assembly: I) polyphenols, II) quinones, III) monosaccharides, IV) saponins, V) alkaloids, VI) phytosterols, and VII) terpenoids, and systematically summarize the common structures related to self‐assembly within each of these seven categories, along with the characteristics of their respective self‐assembly strategies. In addition, the development, advantages, and limitations of NPHM, as well as the characteristics, supramolecular interactions, and preparation methods of NPHM‐based supramolecular bioactive materials are summarized. In the final section, a brief overview of biomedical applications as NPHM‐based supramolecular bioactive materials including disease treatment, attenuation, synergism, delivery capability, improved dosage forms, and permeation is also presented. In summary, the NPHM‐based supramolecular bioactive materials is an emerging system deserved further development.  Next, we will discuss the current bottlenecks in the area, as well as potential directions of exploration that could further advance our understanding and application of the field.

### 6.1 Challenges and Obstacles in the Field of NPHM Self‐Assembly

The main difficulties and limitations currently faced by this field include the following two aspects.

The first challenge is the discovery of NPHM self‐assembly. Whether NPHMs can self‐assemble to form supramolecular products often relies on repeated experiments for three main reasons. First, the complex and diverse chemical structures of NPHMs result in thousands of NPHM exhibiting different self‐assembly behaviors. Second, current detection methods are limited, and no technology exists that can directly prove the entire process of NPHM self‐assembly. Currently, we can only infer the formation process of self‐assembly products through the formation of self‐assembly products, characterization of non‐covalent bonds, and molecular dynamics simulations. Third, there is a lack of systematic summarization and presentation of the self‐assembly principles of this system, which this review aims to explore and summarize.

The second core challenge is to expand the application boundaries of NPHM‐based supramolecular products. Keeping NPHMs in their pristine state without modifying them at the molecular level helps to prevent alteration of the powerful and diverse pharmacological activities, with the significant advantages of being pure, easy to prepare, rich in bioactivity, and biocompatible. But at the same time, it also sets limitations for pursuing a broader, smarter and higher performance path of material development. But at the same time, it also sets some limitations in the path of pursuing broader, smarter, and higher‐performance materials development. Therefore, the key lies in finding the right balance and innovative breakthrough paths while meeting diverse needs.

### 6.2 Future Directions and Explorations in the Field of NPHM Self‐Assembly

1. Further exploration of the self‐assembly laws of NPHM: NPHM possess a diverse range of functional groups and exhibit distinct spatial structures, thereby facilitating intricate non‐covalent interactions that give rise to various self‐assembly mechanisms and behaviors. However, currently, the discovery of self‐assembled products derived from NPHM heavily relies on experimental serendipity. Thus, an in‐depth study of the relationship between structure, functional groups and assembly modes would be beneficial in predicting molecular assembly results. This may result in cost savings and the development of a strong theoretical framework that provides a solid foundation for extensive future research.
2. Attempting diverse, multi‐targeted component co‐assembly: NPHM have the ability to co‐assemble with a variety of molecules with different functions to form supramolecular materials, such as co‐assembled nanoparticles, co‐assembled hydrogels, etc., to exert synergistic therapeutic effects. With practical clinical applications in mind, researchers can skillfully design and conceptualize treatments for specific pathological processes of various diseases and try to assemble and match various functional molecules to achieve multi‐component, multi‐target, and multi‐pathway synergistic treatments.
3. Aiming for clinical translation: The clinical translational value of biomedical materials is a crucial objective. NPHM‐based bioactive materials demonstrate clear advantages for clinical translation, particularly in biodegradability, biocompatibility, cost‐effective manufacturing, manageable process complexity, effectiveness in disease treatment, reproducibility, and environmental friendliness. Translational research on NPHM‐based bioactive materials should be advanced with practical needs in focus. For example, the stability and durability of NPHM‐based supramolecular bioactive materials need to be further improved. A typical example is NPHM‐based hydrogels, which possess advantages such as self‐healing ability, thermal reversibility, and injectability, yet still face the challenge of limited mechanical properties. Optimizing the performance of NPHM supramolecular bioactive materials and elevating their application value constitute pivotal steps toward clinical translation. However, it is imperative to emphasize that this endeavor should adhere to the principle of maintaining the advantageous role of NPHM supramolecular materials.
4. Enriching intelligent application scenarios of NPHM self‐assembly products: No artificial modification, synthetic materials added, just pure use of NPHM to build the self‐assembly system despite the characteristics of simple preparation, but in the intelligent controlled drug release and precise active targeting need to be further in‐depth attempts. For instance, the multi‐stimulus response release system under specific conditions such as light, temperature, pH, electricity, enzyme, etc., and the construction of drug delivery system targeted at target tissues, target organs and target cells. In addition, the application direction can be broadened in multiple dimensions to fully and creatively utilize its more functionality as a smart new material.
5. Providing new perspectives and interpretations for elucidating traditional Chinese medicine (TCM) theories: Many well‐studied NPHM, including artemisinin,^[^ ^257^ ^]^ curcumin^[^ ^258^ ^]^ and so on, are sourced from TCM with a long history of therapeutic use. In response to a patient's unique symptoms, practitioners of Chinese medicine combine a variety of herbs in accordance with a precise summary of the properties of each herb, taking into account each other's priorities, balances, and limitations; this results in the formation of a “formula” which will be given to the patient as a whole for therapeutic purposes. The aforementioned notion of integrating compounds with varying efficacy into a unified entity with a designated purpose in a particular manner and in accordance with a particular scheme coincides with the self‐assembly principle underlying NPHM. Ingeniously, modern research results show that there is indeed a considerable amount of small molecule self‐assembly in both herbal tonics and residues.^[^ ^259^ ^]^ This closely links self‐assembly to TCM, and even the principle of self‐assembly may offer a modern scientific explanation for TCM theories. Moreover, TCM has accumulated a great deal of experience and rules on how natural herbs can be combined to exert specific therapeutic effects over thousands of years of practice, which may also provide hints and inspirations for the self‐assembly of NPHM. For example, the discovery that the two‐component self‐assembly product of aristolochic acid and berberine can significantly reduce the toxicity of aristolochic acid was inspired by TCM experience in the collocation of herbal medicines.^[^ ^17^ ^]^ Consequently, this approach of integrating Eastern and Western perspectives, traditional and contemporary knowledge, experiential wisdom and scientific inquiry, holds potential for further exploration and discovery.

## Conflict of Interest

The authors declare no conflict of interest.
